# Theory of Formation of Polymer Crystals with Folded Chains in Dilute Solution

**DOI:** 10.6028/jres.064A.007

**Published:** 1960-02-01

**Authors:** John I. Lauritzen, John D. Hoffman

## Abstract

A detailed interpretation of the kinetics of homogeneous nucleation and growth of crystals of a linear homopolymer from dilute solution is given. The probability of forming both nuclei with folded chains, and conventional bundlelike nuclei, from dilute solution is analyzed. It is predicted that at sufficiently high dilution, critical nuclei of length $l_{p}^{*}$ will be formed from single polymer molecules by sharp folding of the chain backbone. The step height of the nucleus is given approximately by $l_{p}^{*}=4\sigma_{e}/\Delta f$. Here ***σ****_e_* is the free energy required to form a unit area of the loop-containing end surfaces, and **Δf** is the free energy difference per unit volume of crystal between the crystalline and solution states. The quantity **Δf** is approximately proportional to the degree of supercooling **ΔT**. The growth of these nuclei is then analyzed. After growth, the resulting crystal is flat and platelike, the loops formed by the chain folds being on the upper and lower surfaces. Kinetic factors determine that the distance between the flat surfaces in the grown crystal will vary over only a narrow range about a value that is in the vicinity of 1*=4**σ***_e_*/**Δf**. (Neglecting effects due to edge free energies, the theoretical upper and lower limits are 1*=4**σ***_e_*/**Δf** and 1*=2**σ***_e_*/**Δf**, respectively.) In some cases the predicted temperature dependence of the step height of the grown crystal, 1* = const./**ΔT**, may be modified by the existence of a constant term resulting from the presence of an edge free energy ***ϵ****_p_*. A grown loop-type crystal is predicted to be stable in comparison with a bundlelike crystal of the same shape and volume in a sufficiently dilute solution. The logarithm of the nucleation rate is approximately proportional to 1/(**ΔT**)^2^ near the melting point. The exponent *n* in the free growth rate law is predicted under various assumptions. To the extent that comparison is possible, the predictions given agree with the experimental results obtained by Keller and O’Connor and others on single crystals of unbranched polyethylene grown from dilute solution.

A survey is given of homogeneous nucleation in bulk polymers, where the conventional bundlelike nucleus containing segments from many different molecules is valid, and the essential results compared with those calculated for the dilute solution case.

The theory given for loop nuclei is both general and precise enough at the critical points to suggest that, on crystallization from sufficiently dilute solution, crystals of a definite step height are commonly to be expected for other crystallizable linear polymers than polyethylene, provided loop formation is sterically possible.

## 1. Introduction

Recently, a number of investigators [1, 2, 3, 4],^1^ have prepared single crystals of high molecular weight linear polyethylene by precipitation from dilute solution through supercooling. As observed with an electron microscope, these crystals are shaped like flat parallelepipeds, and the X-ray studies of Keller [1, 2] show that the polymer chains are oriented perpendicular to the flat surfaces. The separation of the flat surfaces is nominally about 120A, and is sufficiently well defined to produce fourth-order reflections with low angle X-rays. The separation of the flat surfaces, which for convenience will be called the “step height,” actually depends on the crystallization temperature, the step height being distinctly smaller at low crystallization temperatures than it is at high ones. Since the mean length of the polyethylene molecules is far in excess of 120A, Keller has proposed that the polymer molecules must be sharply folded in the crystals; the loops resulting from these folds form the two flat surfaces of the platelike crystals.

There appears to be no simple alternative to the initially somewhat startling proposal that the single crystals observed involve chain folding, and we believe that Keller’s hypothesis may be accepted. Keller has indicated that the idea of chain folding in polymers is not entirely new, and refers to an earlier suggestion due to Storks [5].

The objective of this paper is to present a theoretical account of how polymer crystals with chain folds are formed in dilute solution, and why they have the properties they do. It will emerge that crystals with chain folds arise in dilute solution because a primary (homogeneous) nucleus of this type is on kinetic grounds the most likely to appear. Once such a nucleus is formed, it can be shown that the subsequent two-dimensional growth will closely follow the pattern established by the primary nucleus. Thus, the basic reasons such crystals form is to be found in the kinetics of nucleation and growth.

The situation is quite different for homogeneous nucleation in a highly crystallizable bulk polymer. First, the primary (homogeneous) nucleus in bulk polymers is thought to be formed by an alinement of segments of different polymer chains to form a bundlelike nucleus without folds [6, 7], and second, the mean crystallite size in a semicrystalline bulk polymer that has not reached its equilibrium crystallite size distribution (a very difficultly achievable state by any account) is determined largely by the nature of impingements and chain entanglements, and possibly certain strain effects, together with the kinetics of nucleation and growth [6]. (The particular type of strain meant here is that which becomes increasingly great with radial growth.) Eventually, of course, the metastable distribution of crystallite sizes resulting from impingements will change as the impingements relax, and other mechanisms take place, and the equilibrium distribution with large crystallites will be slowly approached, but this does not alter the fact that impingements, entanglements, and possibly strain play an important, if not dominant, role in determining the crystallite size in bulk polymers as they are ordinarily found in the semicrystalline state. Impingements and entanglements, play no important role in impeding the crystallization in dilute solution.

In order to provide a clear development of the theory of crystallization of chain molecules from dilute solution, it is necessary first to bring out some general points connected with homogeneous nucleation theory. At the same time, it is advantageous to mention certain general features of homogeneously induced crystallization in bulk polymers.

## 2. Homogeneous Nucleation and Crystal Growth in Bulk Polymers

### 2.1. Homogeneous Nucleation in Bulk Polymers

According to Turnbull and Fisher [8], the equilibrium rate of homogeneous or primary nucleation in a supercooled bulk phase may be written as

$$I=\frac{NkT}{h}e^{-\Delta F^{*}/kT_{e}-\Delta \phi_{p}^{*}/kT}$$ (1)

where *N* is Avogadro’s number, *k* Boltzmann’s constant, *h* Planck’s constant, *T* the absolute temperature, $\Delta F_{p}^{*}$ the free energy of activation of the supercooled-liquid—nucleus interface, and $\Delta \phi_{p}^{*}$ the free energy of formation of a primary (homogeneous) nucleus of critical size. In eq (1)*I* is in nuclei·mole^−1^·sec^−1^. The quantity $J=\left(kT/h\right)\exp\left[\Delta F_{p}^{*}/kT\right]$, which is the jump rate in events per second at the interface, may be written as $\left(kT/h\right)\exp\left[\Delta S_{p}^{*}/k-\Delta H_{p}^{*}/kT\right]$, where $\Delta S_{p}^{*}$ is the entropy of activation, and $\Delta H_{p}^{*}$, the enthalpy of activation. For a polymer, it may be assumed that the smallest unit that may attach to the embryo or nucleus in an elementary process is a small segment of molecular weight *M* and length *l_0_*. Hence we may write eq (1) in the form

$$I=I_{0}e^{-\Delta H_{p}^{*}/kT_{e}-\Delta \phi */kT}$$ (2)

where *I*_0_ is $\left(NkT/hM{\bar{V}}_{l}\right)\exp\left(\Delta S_{p}^{*}/k\right)$ which has the units nuclei·cm^−3^·sec^−1^. The quantity ${\bar{V}}_{l}$ is the specific volume of the supercooled liquid at the temperature of crystallization. The main item of interest here is the form of $\Delta \phi_{p}^{*}$ for bulk polymers. The Turnbull-Fisher equation is derived on the assumption that many elementary steps are required to reach $\Delta \phi_{p}^{*}$.

In a bulk polymer, it is commonly assumed that the nucleus is bundlelike, and is formed through the alinement of segments of different polymer chains [6, 7]. This hypothesis certainly seems plausible for a bulk polymer, and can be used to give a detailed interpretation of the rate of injection of primary nuclei in a bulk polymer.

Two general types of bundlelike primary nuclei must be considered. The first of these is one where there is no minimum restriction on the length, or the number of segments contained in its cross-sectional area. Calculations for this nucleus yield results that are valid in a temperature range near the melting point, region *A.* The second is a nucleus where the length is restricted to *l*_0_ (which is the length of a segment), but where the number of segments in the cross section is still unrestricted. Results obtained for this nucleus are valid in a temperature range, region *B*, that extends from somewhat below the melting point to a temperature that is considerably lower. A discussion of the properties of these two types of bundlelike nuclei has been given in an earlier publication [6], and what is given below is intended mainly as a summary. At still lower temperatures, region *C* type nucleation will prevail, and this will be brought into the discussion at the proper place.

#### Region A

**Figure 1:**
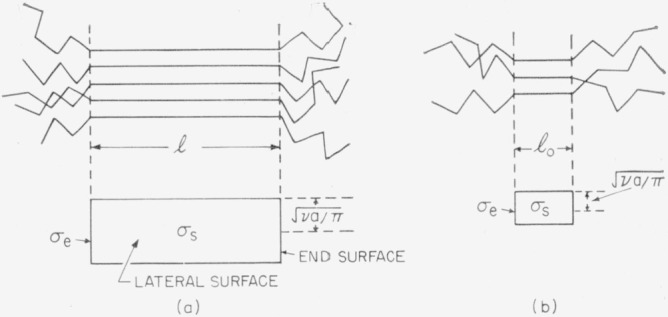
Homogeneous bundlelike nucleus (bulk polymer). (a) Nucleus of length *l* and “radius” $\sqrt{va/\pi }$ valid in region A, where there is no minimum restriction on *l* or *v.* (b) Nucleus of fixed length *l*_0_ and “radius” $\sqrt{va/\pi }$ valid in region *B.*

Consider first the nucleus with unrestricted length and cross-sectional area. The model used is illustrated in figure 1*a.* For this nucleus, the free energy of formation may be written in a general way as

$$\Delta \phi_{p\left(A\right)}=2va\sigma_{e}+C\sqrt{va}l\sigma_{s}-val\Delta f.$$ (3)

Here *v* is the number of segments in the cross section of the nucleus, *a* the cross-sectional area of a segment, *l* the length of the nucleus, *C* a numerical constant that depends only on the shape of the cross section, and Δ*f* the free energy difference per unit volume of crystal between the supercooled liquid and the crystal. The quantity *va* is the area of the end of the nucleus or embryo. The quantity *σ_s_* is the work required to form a unit area of the lateral surface from the crystal, and *σ_s_* is the corresponding work for the end of the crystallite. If at any given degree of supercooling *v* and *l* are increased, Δ*ϕ_p_*_(_*_A_*_)_ goes through a maximum where it has the value $\Delta \phi_{p\left(A\right)}^{*}$ and then falls rapidly through zero to strongly negative values, the latter implying increasing stability with increasing size. The critical values of *l* and *va* can readily be determined by setting (∂Δ*ϕ_p_*_(_*_A_*_)_/∂*l*)*va* and ${\left(\partial \Delta \phi_{p(A)}/\partial \sqrt{va}\right)}_{l}$ equal to zero. Thus,

$$l*=\frac{4\sigma_{e}}{\Delta f},$$ (4)

and

$$\left(va\right)*=\frac{C^{2}\sigma_{s}^{2}}{{\left(\Delta f\right)}^{2}}.$$ (5)

Substitution of eqs (4) and (5) into (3) yields the result

$$\Delta \phi_{p\left(A\right)}^{*}=\frac{2C^{2}\sigma_{s}^{2}\sigma_{e}}{{\left(\Delta f\right)}^{2}}.$$ (6)

Thus, in region *A*, where both *l* and *va* are not subject to a minimum restriction, the rate of homogeneous nucleation is^2^

$$I_{A}=I_{0}e^{-\Delta H_{p}^{*}/kT}e^{-2C^{2}\sigma_{s}^{2}\sigma_{e}/{\left(\Delta f\right)}^{2}kT}$$ (7)

In this expression

$$C=2\pi^{\frac{1}{2}}$$ (8)

for a cylindrical nucleus, and

$$C=\frac{2}{\sqrt{\sin\;\psi }}\frac{x+y}{\sqrt{xy}}$$ (9)

for a nucleus where the cross section is a parallelogram with sides *x* and *y*, and apex angle *ψ*. The quantity (*va*)* is related to the square of the “radius” of the critical-sized nucleus.

**Figure 2:**
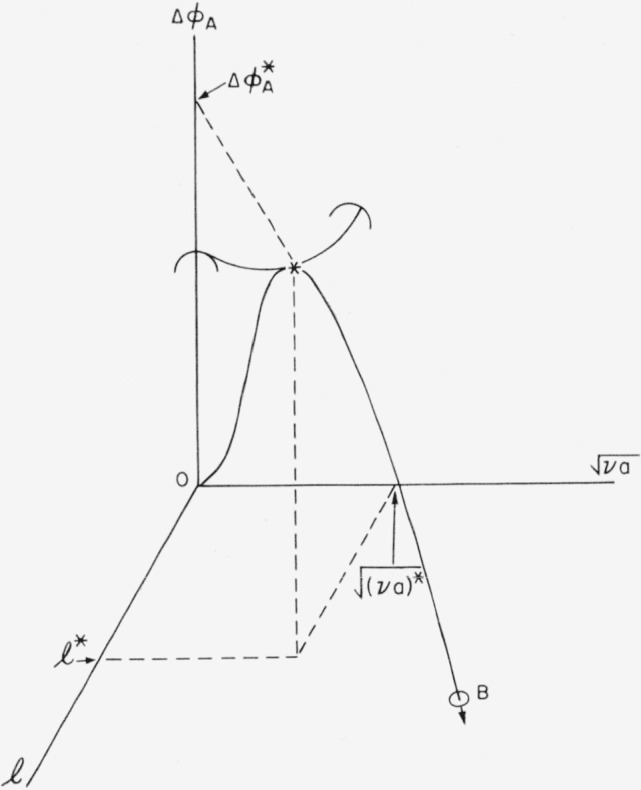
Free energy surface for formation of a critical-sized homogeneous bundlelike nucleus for bulk polymers in region A. The reaction path is the heavy line 0–∗–*B.* The nucleus is of critical size at the saddle point marked ∗. The free energy surface for region B is similar, except that l*=*l*_0_.

For a strictly cylindrical nucleus, $r*={\left[\left(va\right)*/\pi \right]}^{\frac{1}{2}}=2\sigma_{s}/\Delta f$, and $\Delta \phi_{p\left(A\right)}^{*}=8\pi \sigma_{s}^{2}\sigma_{e}/{\left(\Delta f\right)}^{2}$, results that have been given previously [6, 9]. The reaction path on the free energy surface described by eq (3) for the formation of the critical-sized nucleus is shown in figure 2. The critical-sized nucleus of length l^*^ and “radius” ${\left[\left(va\right)/\pi \right]}^{*\frac{1}{2}}$ is indicated by an asterisk, and the reaction path is designated by the heavy line O–∗–*B.* The point * is at a saddle point in the free energy surface. The embryo grows into a nucleus and thence into the stable region (which is below the $l-{\left[va\right]}^{\frac{1}{2}}$ plane) by both lengthwise and “radial” growth.

#### Region B

For a bundlelike nucleus, it is necessary to recognize that *σ_e_* might possibly be considerably smaller than *σ_s_.* As one traces the environment of the various segments from the interior of the crystal out through the lateral surface into the liquid phase, a sharp and quite large drop in the degree of order will be noticed just at the crystal surface. Thus, the value of *σ_s_* will correspond reasonably closely to the surface free energy for a nonpolymeric molecular crystal of the same chemical type, and will commonly lie in the range 5 to 25 erg·cm^−2^. On the other hand, the drop in degree of order as one traverses a path from the center of the crystal out through the end will not be as sharp as in the case above. Because of this fact it seems plausible to suppose that *σ_e_* will in some polymers be rather smaller than *σ_s_.* However, *σ_e_* cannot be zero, since this would imply no difference in free energy between the end of the crystallite or nucleus and the supercooled liquid.

The significance of the fact that *σ_s_* may be considerably larger than *σ_e_* for the bundlelike nucleus characteristic of primary nucleation in region *A* is that l*, as given by eq (4), may, at some temperature *T_c_* that is not too far below the melting point, fall close to the irreducible segment length, *l*_0_. In this case, *l* must not be treated as a variable near and below *T_c_.* Using the relation [10]^3^

$$\Delta f=\frac{\Delta h_{f}T\Delta T}{T_{m}^{2}},$$ (10)

where Δ*h_f_* is the heat of fusion at the equilibrium melting temperature, *T_m_*, and Δ*T=T_m_−T*, where *T* is the isothermal crystallization temperature, it is found to a sufficient approximation that

$$\Delta T_{c}=\frac{4T_{m}\sigma_{e}}{l_{0}\Delta h_{f}}.$$ (11)

Here Δ*T_c_* is the degree of supercooling that corresponds to the onset of region *B.* At lower temperatures, we must consider a primary nucleus with fixed length *l*_0_, and variable *va*, as shown in figure 1b. In this case we have

$$\Delta \phi_{p\left(B\right)}=2va\sigma_{e}+C\sqrt{va}l_{0}\sigma_{s}-val_{0}\Delta f$$ (12)

which leads to

$${\left(va\right)}_{B}^{*}={\left[\frac{Cl_{0}\sigma_{s}}{2\left(l_{0}\Delta f-2\sigma_{e}\right)}\right]}^{2}$$ (13)

and

$$\Delta \phi {*}_{\left(B\right)}=\frac{C^{2}l_{0}^{2}\sigma_{s}^{2}}{4\left(l_{0}\Delta f-2\sigma_{e}\right)}.$$ (14)

In region *B* (or more precisely, from somewhat below *T_c_* on down to considerably lower temperatures) the condition *l*_0_Δ*f*>>2*σ_e_* may be expected to hold. With this, eq (14) reduces to the simple form

$$\Delta \phi_{p\left(B\right)}^{*}≅\frac{C^{2}l_{0}\sigma_{s}^{2}}{4\Delta f},$$ (15)

and the rate of primary nucleation becomes

$$I_{B}=I_{0}e^{-\Delta H_{p}^{*}/kT}e^{-C^{2}l_{0}\sigma_{s}^{2}/4\Delta fkT}.$$ (16)

The values of *C* are the same as those given for region *A*; for the particular case of a strictly cylindrical nucleus, $\Delta \phi_{p\left(B\right)}^{*}$ is $\pi l_{0}\sigma_{s}^{2}/\Delta f$ [6].

Equations of the general form of (15) and (16) have sometimes been sharply criticized, apparently because of the incorrect belief that they could be derived only on the basis that *σ_e_*≡0, the latter being generally conceded to be impossible. However, the derivation sketched above makes it perfectly clear that eqs (15) and (16) hold if *l*_0_Δ*f*>>2*σ_e_*, and there is no implication that *σ_e_*≡0 [6].

Region *B* type primary nucleation will prevail down to a temperature *T_cc_* corresponding to a degree of supercooling of approximately

$$\Delta T_{cc}≅\frac{C\sigma_{s}T_{m}}{2\Delta h_{f}\sqrt{{\left(va\right)}_{0}}}.$$ (17)

In the case that *σ_s_>>σ_e_*, Δ*T_cc_* will be larger than Δ*T_c_*, with the result that region *B* will cover a substantial range of temperature.

The free energy surface described by eq (12) has a saddle point at l*=l_0_ and *va*=(*va*)*. Thus, both the embryo and nucleus always have a length *l*_0_, but once of stable size, there is no inherent restriction on the addition of segments to increase the length. Lengthwise growth is in fact certain to occur [6]. Such a nucleus will increase in size by appropriate growth mechanisms until stopped by impingements or other factors (see sec. 2.2).

#### Region C

At crystallization temperatures below *T_cc_*, the “radius” of the primary nucleus, [(*va*)_0_]^1/2^, will be close to the size of the unit cell, i.e., it will contain roughly 5 to 7 segments. While this radius is not irreducible in a strict sense, the small size of the stable nucleus below *T_cc_* will lead to an excess number of nuclei owing to the fact that embryos of this size in the superheated state will be carried down in the supercooling process to the supercooled state. This will cause an enhanced rate of crystallization compared with region *B* or *A.* In the particular case where *σ_e_* is larger than envisioned previously, and exceeds *l*_0_*Cσ_s_*/4[(*va*)_0_]^1/2^, which is *l*_0_*σ_s_/*2*r*_0_ for a cylindrical nucleus of radius *r*_0_, region *B* will be absent, and the system will go directly from region *A* type homogeneous nucleation to that characteristic of region *C*.

Several important points concerning the nature of homogeneous nucleation in bulk polymers may now be emphasized. The first is that two types of temperature dependence are to be expected for the rate of nucleation. Sufficiently near the melting point, i.e., in region *A*,

$$\ln\frac{I_{A}}{I_{0}}=-\frac{\Delta H_{p}^{*}}{kT}-\frac{\alpha }{T^{3}{\left(\Delta T\right)}^{2}},$$ (18)

where the constant *α* is $2C^{2}\sigma_{s}^{2}\sigma_{e}T_{m}^{4}/\Delta h_{f}^{2}k$. This is the same general form as is exhibited by nonpolymeric systems. At moderate to high degrees of supercooling, region *B*, the temperature dependence is

$$\ln\frac{I_{B}}{I_{0}}=-\frac{\Delta H_{p}^{*}}{kT}-\frac{\beta }{T^{2}\Delta T},$$ (19)

where the constant *β* is $C^{2}l_{0}\sigma_{s}^{2}T_{m}^{2}/4\Delta h{\;}_{f}k$. Equation (19) is a special result in that it reflects the segmental nature of the polymer chain, having been derived assuming *l*_0_ was a constant. *In the special case where σ_e_*≧*l*_0_*σ_s_*/2*r*_0,_ *region B will be absent, and the system will exhibit a temperature dependence of the form of* eq (18) *down to the A→C transition*. However, in some cases it is to be anticipated that *σ_e_* will be sufficiently less than *σ_s_* to cause region *B* to make its appearance. Region A will be large if *σ_e_*~*σ_s_*.

Both eqs (18) and (19) lead to a maximum in *I/I*_0_ when plotted as a function of temperature. The terms exp[−*α*/*T*^3^(Δ*T*)^2^] and exp[−*β*/*T*^3^Δ*T*] lead to strongly negative temperature coefficients for the rate of injection of nuclei, but this effect is eventually overwhelmed by the term $\exp\left[-\Delta H_{p}^{*}/kT\right]$ that arises from the jump rate, and which has a positive temperature coefficient. Hence a maximum exists in *I_A_* and *I_B_.*

The second point is that there is nothing in the foregoing which suggests a highly uniform step height of the general character found in crystals formed from dilute solution. The only feature in the theory for bulk polymers that is even slightly suggestive of a pronounced step height, where the long axes of the polymer molecules are in the correct configuration with respect to the crystal surfaces, is the behavior of $l*=4\sigma_{e}T_{m}^{2}/\Delta h_{f}T\Delta T$ in region *A*. However, an unacceptable large value of *σ_e_* has to be introduced to cause l* to be anywhere near as large as is observed for polymer crystals obtained from dilute solution. Furthermore, such a nucleus will certainly grow lengthwise, and it is very difficult to imagine why it would grow to a practically completely uniform length which would correspond to a step height. (More will be said of this later.)

The third point is that in a bulk polymer, the bundlelike nucleus, made up from segments of different polymer chains, is energetically the most favorable that can be conceived. Unless prevented by some factor not yet considered, this is the type of nucleus that should commonly appear in a bulk homopolymer. Then if no special strain effects interfere (say in the radial growth), such nuclei should grow both radially and length wise.

We turn now to some general considerations that have to do with the nature of the growth of the bundlelike primary nuclei, and the effects that cause such growth to cease in bulk polymers, or at least slow down to a marked extent. Once certain general features of the growth process in bulk polymers have been brought out, the discussion of primary nucleation and growth in dilute solution with chain folding can be given.

### 2.2. Crystal Growth, Bulk Rate Constants, and Impingements in Bulk Polymers

Two features of the growth process in bulk polymers are of interest here. The first is that the primary bundlelike nucleus without chain folding can, at least initially, grow radially and lengthwise. Each of these growth mechanisms is nucleation controlled sufficiently near the melting point. The second point is that the growing crystals will impinge on one another in such a manner as to essentially stop or markedly retard lengthwise and radial growth in a manner that can hardly lead to a highly uniform step height of the type found in dilute solution. In the special case where strain limits radial growth (see below), only the distribution of lengths will be impingement controlled, but this will still not correspond to an essentially fixed step height.

Consider first the types of growth that may occur, at least initially, for a bundlelike nucleus. Denote radial growth as *G_r_=dr*/*dt* (where in general $G_{r}\propto d{\left[va\right]}^{\frac{1}{2}}/dt$) and lengthwise growth as *G_l_=dl*/*dt.* Further, define the free bulk growth rate as

$$\chi^{\prime }=Z_{n}\;t^{n}$$ (20)

where χ′ is the mass fraction crystallized, *t* the time, and *n* an exponent that depends on type of nucleation and the mode of growth. The free bulk growth rate is the rate at which the polymer would crystallize if the growing crystals were independent of one another. Values of *n* for various modes of growth with homogeneous initiation (i.e., primary nuclei born sporadically in time) are shown in table 1. The relationship between *Z_n_*, *I*, *G_l_* and *G_r_* are also shown.

The growth mechanism denoted by *G_l_* is to be described by an expression of the general form

$$G_{l}=G_{0}e^{-\Delta H_{g}^{*}/kT}e^{-\gamma /T^{2}\Delta T}$$ (20)

so that

$$\ln\frac{G_{l}}{G_{0}}=-\frac{\Delta H_{g}^{*}}{kT}-\frac{\gamma }{T^{2}\Delta T}$$ (21)

Here *γ* is a constant similar in character to *β*, and $\Delta H_{g}^{*}$ is the enthalpy of activation at the super-cooled-liquid—growth-nucleus interface. The form of eq (20) arises from the fact that in the experimentally accessible region the growth nucleus is characterized by one fixed and temperature independent dimension of molecular size, usually a thickness of one molecule or segment length (circa 2.5 to 20A). However, the temperature dependence of the growth mechanism denoted by *G_r_* may differ from that of *G_l_*, since the secondary nucleus may be of a different nature. In general, both *G_l_* and *G_r_* will go through a maximum below the melting point, and will possess a strongly negative temperature dependence near the melting point. In the event that *σ_s_>>σ_e_*, the radial growth nucleus in the experimentally accessible region may have *two* fixed and temperature independent dimensions of molecular size. The radial growth nucleus will generally be easier to form than the lengthwise growth nucleus, so the condition *G_r_>G_l_* is commonly to be anticipated. (See, however, remarks below concerning possible retardation of radial growth by strain.)

**Figure 3:**
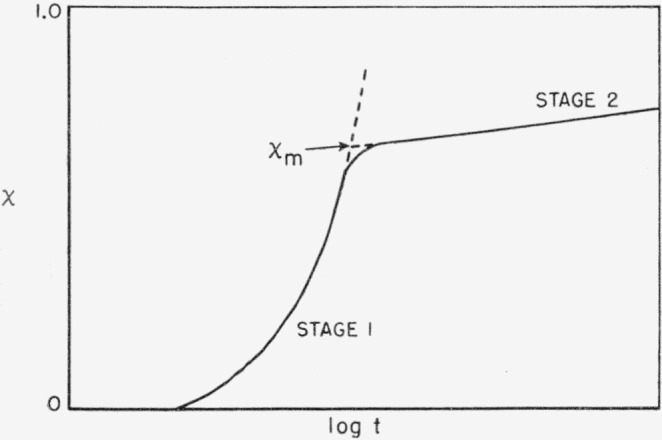
Schematic diagram showing course of crystallization in a bulk polymer. χ is the mass fraction crystallized and *t* the time. Stage 1 strongly reflects the free growth rate χ*' = Z_n_t*^n^. The pseudoequilibrium degree of crystallinity is denoted χ*_m_*, and is the result of impingements and entanglements. Stage 2 slowly carries the crystallization beyond χ*_m_.*

We must now ask what processes retard the free growth rate of the crystals in a bulk phase. Impingements and entanglements are certainly important factors [6]. The growing crystallites will run into each other, entanglements will occur in the vicinity of such “collisions”, and this will tend to stop growth. The retardations due to impingements are relatively weak early in the crystallization, but gradually get stronger. The isotherms in this range, which is called “stage 1,” will commonly be superposable simply by shifting the time scale [6]. Estimates of the free bulk growth rate constant, *Z*, may be obtained by analysis of stage 1 data. However, the system will approach a degree of crystallinity, well short of complete crystallization, where there is a massive degree of impingement (fig. 3). We refer to this as the pseudoequilibrium degree of crystallinity, χ*_m_*. Detailed theoretical calculations due to Lauritzen [11], and certain experimental studies [6], fully justify the view that impingements will lead to the effect indicated. Near and above χ*_m_*, the crystallization process is exceedingly slow. Other workers have called this “secondary crystallization” but for convenience we have termed it “stage 2.” Relaxation of impingements and entanglements to form crystallites with greater length and radii is one of the principal crystallization processes in stage 2. The equilibrium degree of crystallinity is thus approached very slowly due to the intercession of a massive degree of impingement at χ*_m_*.

After the stage 2 mechanism has pursued its course for a sufficient time, the length and radius of a few of the crystallites will be large enough to melt quite close to the equilibrium melting temperature, *T_m_.* In the vicinity of χ*_m_*, the crystallites will often be rather small, and impingements will have set up a distribution of crystallite sizes. These effects will cause rather broad and low melting. The particular distribution that prevails at χ*_m_* changes only very slowly toward the equilibrium one. Neither the distribution of radii and lengths resulting from impingements, nor even the true equilibrium one, is consistent with a uniform step height.

Another effect that may subdue growth of bundlelike nuclei is strain. Thus, while bundlelike *nuclei* may form easily, radial *growth* to large size may be hindered by the strain that results from the mismatch of the segments in the crystal with those in the “liquid” just outside the ends. Such a situation could be treated theoretically in terms of a *σ_e_* value that increased with *v.* The effect mentioned could conceivably severely restrict radial growth of bundlelike nuclei in some cases, causing a nearly constant crystallite radius to be observed. However, the stoppage of lengthwise growth will in such a case still be controlled by impingements, and not correspond to a step height of the type found in folded crystals.

Much of what has been said concerning the nature of impingements may be found in more detail in a previous article [6].

## 3. Homogeneously Induced Crystallization of Polymer From Dilute Solution

### 3.1. Preliminary Analysis of Homogeneous Nucleation From Dilute Solution

In order to set the stage for the detailed analysis to follow in subsequent sections, an elementary analysis of the problem of nuclei with chain folding is given first. This has the advantage of permitting an early emphasis on the simple physical picture involved, and has the virtue of clearly indicating just what points must be subjected to more searching analysis.

When a polymer is dissolved at high dilution in a relatively good solvent, the polymer molecules tend to be essentially isolated from each other. If the solution is supercooled, the polymer will tend to crystallize from the solution. The kinetics of this crystallization will be governed by the nucleation and growth process. Since the polymer molecules are essentially isolated from one another, the primary nucleus will tend to be formed, if at all possible, from a single polymer molecule. The formation of these nuclei is treated below and it will be shown that in sufficiently dilute solution these nuclei, characterized by chain folding, are kinetically favored over bundlelike nuclei containing segments from many molecules of the type discussed in the previous section for bulk phases. This treatment explains the main features of the single crystals obtained by Keller and others, and predicts other properties which should be capable of verification.

**Figure 4:**
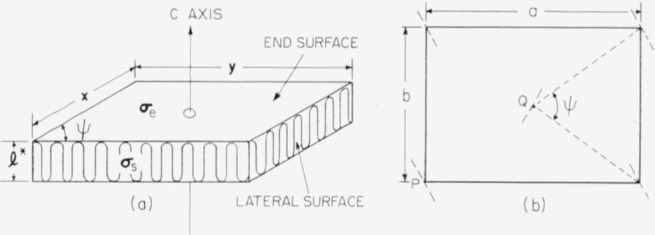
Details of loop-type polyethylene crystals formed from dilute solution. (a) Polyethylene crystal with step height l* showing orientation of chains. (b) View of unit cell along *c*-axis showing orientation of ribbonlike hydrocarbon chain---.

We shall outline in some detail the characteristics of the single crystals of polyethylene prepared from a dilute solution of xylene [1, 2]. These crystals, as revealed by electron micrographs, are flat parallelepipeds which are shown schematically in figure 4a. The step height, l*, was measured by low angle X-ray scattering, and reflections up to the fourth order were observed. The step height increased from 90 to 140 A with increasing crystallization temperature. The polymer chains lie approximately perpendicular to the two large flat faces of the crystal, i.e., parallel to the *c*-axis in figure 4a. The loops formed by the folding of the polymer molecules form the two flat surfaces of the crystal. In figure 4b the crystal is shown as viewed along the *c*-axis. The polymer chains intersect the plane normal to the *c*-axis at the corners and at the center of the rectangle. The planes determined by the two rows of carbon atoms in the zig-zag polymer chain backbone are shown as triple dashed lines. It has not been definitely determined which chains in figure 4b are connected by the loops, but Keller has indicated that it is sterically possible for the chains at *P* and *Q* in the figure to be connected by a loop containing three to five carbon atoms. The arrangement of the chains shown in figure 4b is essentially that given by Bunn [12].

In the discussion of the nucleus with folds the following definitions are employed. First, ***v*** is taken to be the number of segments in the cross-sectional area of the nucleus or embryo, and **a** is the cross-sectional area of each segment. The area of the end of the nucleus is ***v*a**. The length of the nucleus or embryo is designated **l***_p_.* All of these definitions are analogous to those used earlier for the bundlelike nucleus. Refer to the set of segments comprising the length of a nucleus or embryo, **l***_p_*, as a *step element*; the step element length includes the (small) length involved in the folds at either end. The number of step elements in a nucleus is equal to ***v***, and the total number of folds is equal to ***v***−1.

**Figure 5:**
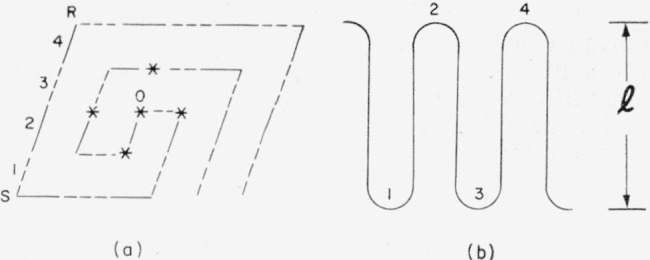
Loops in homogeneous nucleus formed from dilute solution. (a) View of proposed loop nucleus along *c*-axis. Loop facing reader—, loop away from reader. The cross marks near 0 show one unit cell (Cf fig. 4b). (b) Cut through plane *RS* showing odd-numbered loops (down) and even-numbered loops (up).

We now introduce a particular model of the polyethylene crystal in order that we may have a specific picture in mind while calculating the properties of crystals formed by the folding of polymer chains. This model, which is essentially that suggested by Keller and O’Connor, is shown in figure 5. A single molecule forms the crystal through folding of its backbone as it progresses outward in a double spiral from a central position 0. (At a later stage in the development of the crystal, other molecules may, of course, participate.)

The above model of the nucleus with a double spiral is only one of several possibilities, but it still embodies the important general characteristics of nuclei with chain folding. These characteristics apply not only to polyethylene but also to any polymer that can form such nuclei. First, it is possible to form nuclei from a single polymer molecule. Second, the crystals formed through chain folding possess sharp and definite boundaries between crystalline and noncrystalline regions. This is in contrast with the end surface of crystallites discussed in the section on bulk polymers. Third, a change in any reasonably short period of time^4^ of the step height requires the melting (or dissolving) of the crystal and recrystallization with a new “step height”. Fourth, if a molecule has formed an array of ***v*** step elements, the ***v***+1st step element may be added simply by the folding of a free end (or ends) of the polymer molecule. Fifth, when a polymer molecule forms an array of ***v*** parallel step elements there will be ***v***−1 folds in the nucleus. It is emphasized that all five of these items hold for either a double spiral model, a single spiral model (not shown), or any of a number of other configurations.

The rate of formation of nuclei constructed from a single polymer molecule through chain folding will be calculated by a procedure very similar to that used in section 2. Bold faced symbols are used for many of the quantities involved in order to clearly differentiate them from those pertaining to the conventional bundlelike nucleus described earlier. The free energy relative to the solution state of a primary nucleus composed of ***v*** step elements of length **1** may be written as

$$\Delta \phi_{p}=2va\sigma_{e}+C\sqrt{va}l\sigma_{s}+2C\sqrt{va}\epsilon_{p}-val\Delta f,$$ (22)

where **a** is the cross-sectional area of a segment in the crystal, *C* is a numerical factor depending upon the shape of the nucleus, and **Δf** is the free energy difference per unit volume of crystal between the polymer in the supercooled solution and the crystal. The quantity ***σ****_s_* is the work required to form a unit area of the lateral surface from the crystal and ***σ****_e_* the corresponding work for the end of the crystal. The quantity ***ϵ****_p_* is the work required to form a unit length of “edge” from the crystalline phase.

The relative size of ***σ****_e_* and ***σ****_s_* may be estimated from the following considerations. Both the lateral and end surfaces of the nucleus with folds present an abrupt change from crystalline order with respect to the solution. In addition, on the end surface, an amount of work **q** kcal/mole of loops will be required to form a fold. When there are ***v*** segments in the cross section of the nucleus, there will be ***v***−1 folds, and area of the two ends is 2 ***v*a**. Then we have

$$\sigma_{e}=\sigma_{eo}+\frac{q\left(v-1\right)}{2va}≅\sigma_{eo}+\frac{q}{2a},$$ (23)

where ***σ****_e_*_o_ represents the (probably small) contribution to ***σ****_e_* above that of fold formation. We should expect to find **q** with a value on the order of magnitude of 1 kcal/mole of loops.^5^ In making this rough estimate, it was assumed that the principal contribution to **q** was the energy required to bring the part of the polymer chain in the folds (ca. five carbon atoms in the case of polyethylene) into the appropriate higher internal rotational states. If **a**=18×10^−16^ cm^2^, and **q**=1 kcal/mole, **q**/2**a**=20 erg·cm^−2^. We expect no really large difference between ***σ****_s_* and *σ_s_*, the lateral surface free energies of the nuclei with loops, and the bundlelike nuclei, respectively. The important differences in surface free energy between bundlelike nuclei, and nuclei with loops, can be summarized in the following way. For the bundlelike nucleus we have

$$\sigma_{s}≳\sigma_{e},$$ (24)

where *σ_s_* is a “normal” value, usually in the range 5 to 25 erg·cm^−2^. For nuclei with loops, we have instead

$$\sigma_{e}>\sigma_{s},$$ (25)

which is in sharp contrast to (24). Noting that ***σ****_s_* will ordinarily have a “normal” value, we may effect the comparison between the loop and bundlelike types of primary nuclei by writing

$$\sigma_{s}≅\sigma_{s}$$ (26)

and

$$\sigma_{e}>\sigma_{e}.$$ (27)

The quantity **Δf** in eq (22) may be approximated by [13]

$$\Delta f=\Delta h_{f}\cdot \frac{\left(T_{m}-T\right)}{T_{m}}=\frac{\Delta h_{f}\Delta T}{T_{m}},$$ (28)

where **Δh***_f_* is the heat of fusion per unit volume of crystal, and **T***_m_* is the equilibrium melting temperature of the crystal, both in the presence of large amounts of the solvent.

The presence of the edge energy term in eq (22) is not essential for the theory developed in this paper, and the general conclusions drawn about crystals with folds are independent of ***ϵ****_p_*. Since the value of ***ϵ****_p_* will depend on the detailed morphology of the crystals with folds, which is not treated in this paper, and for the reason that its inclusion at this juncture would not elucidate any essential points, it is set equal to zero in the remainder of this section. Nevertheless this term is included in eq (22) for completeness, and the consequences of ***ϵ****_p_* possessing a nonnegligible value will be assessed later.

**Figure 6:**
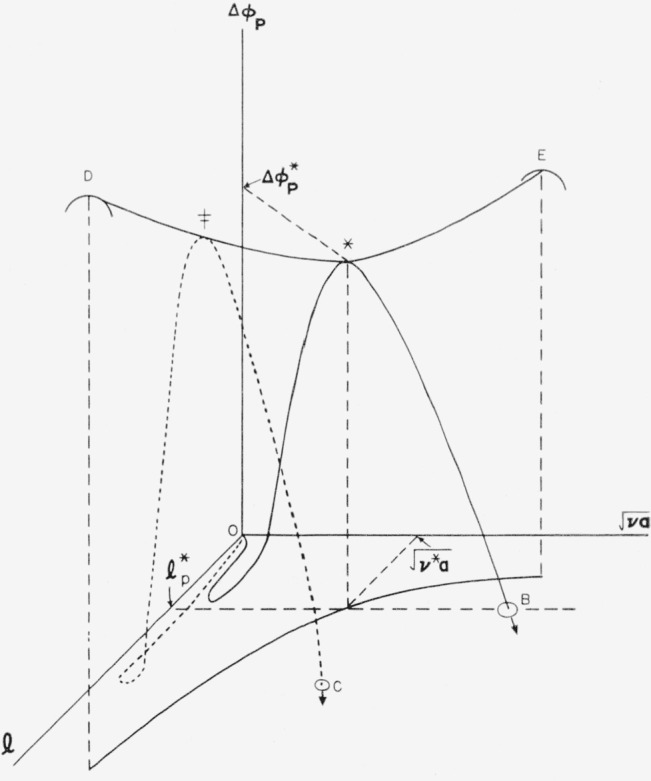
Free energy surface for formation of critical-sized homogeneous nuclei with loops (dilute solution). The heavy line 0–∗–*B* shows most probable reaction path; * is the saddle point across ridge D–E. The dotted line 0⋯‡⋯*C* shows an other possible reaction path across ridge D–E, where $\Delta \phi_{p}^{‡}>\Delta \phi_{p}^{*}$.

The energy surface described by eq (22) is shown in figure 6. It is formally similar to the energy surface for bundlelike nuclei. In both cases the most probable nucleation path passes through the saddle point. The difference between the two types of nuclei is that certain restrictions apply to the paths of nucleation on the surface for nuclei with loops that do not apply to bundlelike nuclei. For nuclei or embryos with folds, the elementary process is the addition or subtraction of a step element. Then the paths by which nuclei with folds are formed are characterized by a length that is invariant as the embryo or nucleus grows. Two paths of nucleation are shown in figure 6. One path passes through the saddle point, while the other path passes over a higher energy barrier. It will be shown subsequently that most of the nuclei formed will pass through or near the saddle point, and will therefore possess a length close to the value at the saddle point, $l_{p}^{*}$. The coordinates of the saddle point may be found by calculating (**∂Δ*ϕ****_p_*/**∂l**)*_v_* and (**∂Δ*ϕ****_p_*/**∂*v*a**)_1_ from eq (22), and equating them to zero. It is found that

$$l_{p}^{*}=\frac{4\sigma_{e}}{\Delta f},$$ (29)

and

$$v*=\frac{C^{2}\sigma_{s}^{2}}{a{\left(\Delta f\right)}^{2}},$$ (30)

which lead in a straightforward manner to the result

$$\Delta \phi_{p}^{*}=\frac{2C^{2}\sigma_{s}^{2}\sigma_{e}}{{\left(\Delta f\right)}^{2}}.$$ (31)

Already from eq (29) we can preceive the origin of a large nucleus length for nuclei with folds as compared with that for bundlelike nuclei. From this expression and **Δf**=**Δh***_f_***ΔT**/**T***_m_*, it is found for nuclei with folds that

$$l_{p}^{*}=\frac{4\sigma_{e}T_{m}}{\Delta h_{f}\Delta T},$$ (32)

whereas from eq (4) and (10) we find, omitting the relatively unimportant factor *T_m_/T*, that for bundlelike nuclei

$$l*=\frac{4\sigma_{e}T_{m}}{\Delta h_{f}\Delta T}.$$ (33)

Since from eq (27), ***σ****_e_*>*σ_e_*, it is seen that $l_{p}^{*}$ should generally be considerably larger than l* under corresponding conditions of supercooling. As will be seen later, our estimate that ***σ****_e_*~20 erg·cm^−2^ leads to values of 1* in the vicinity of 100A at a moderate degree of supercooling. The fundamental reason for the large value of $l_{p}^{*}$ as compared to l* is, of course, the work **q** required to form the fold.

On account of the relatively large value of ***σ****_e_* compared to *σ_e_*, it is to be anticipated that the nuclei formed in the experimentally accessible temperature range for dilute solutions will not ordinarily be subject to a minimal restriction of the type that causes the appearance of region *B* or *C* type nucleation in bulk polymers. Thus, our treatment of nuclei with chain folds is in some respects analogous to region *A* type nucleation in bulk polymers.

Equation (32) shows that $l_{p}^{*}$ should increase as the crystallization temperature increases. Nuclei with lengths greater or less than $l_{p}^{*}$ are improbable for kinetic reasons, as will be brought out subsequently.

It is seen that there is little difficulty in explaining why a nucleus with folds should have fairly large dimensions, corresponding in magnitude to the step height determined by Keller. The really critical issue is why this nucleus of length $l_{p}^{*}$ does not continue to grow in the **1** dimension, but chooses instead to grow in the **x** and **y** dimensions. This question will be pursued in considerable detail later, but it is considered fitting at this juncture to mention the general nature of the arguments showing that the crystal will maintain a length 1* that is close to $l_{p}^{*}$ as it grows. The presence of the folds on the end surfaces prevents rapid growth of the nucleus or embryo in the **1** direction of the simple type that can readily occur for a bundlelike nucleus in its *l* direction. The problem then becomes that of assessing the relative growth rates in the **1** direction, and on the lateral surfaces, for the loop type nucleus. Consider first what happens after a critical-sized nucleus with folds is formed. Since there will likely be very few other polymer molecules close by, the molecule already involved in the nucleus will continue to “crystallize,” forming a primary crystallite containing one molecule. It can be demonstrated that the primary crystallite which on a kinetic basis has the highest probability of formation will in fact possess a length that is close to $l_{p}^{*}$. It is, for example, highly improbable on energetic grounds that a new loop will protrude far above the plane of loops already established. The same is true of the set of new loops in a larger body. A quite similar argument applies to the growth of the crystallite when another polymer molecule enters the picture. Again the energetically least expensive growth nucleus contains a loop, and has a length 1* that is close to $l_{p}^{*}$. Growth on the two primary crystallite faces containing the loops is not impossible, but will be subdued by the circumstance that a secondary or growth nucleus on this surface is nearly as difficult to form as the original primary nucleus. Considerable attention will be paid to the possible variation of the step height as the crystal grows, and this will be shown to be small. The relatively narrow distribution of step heights around the mean value of the step height is related to the nature of the saddle point in the free energy surface describing the rate of nucleation and growth.

In appendix 5.1 it is shown that to good approximation the number of stable nuclei formed isothermally per unit volume of solution per unit time is

$$I=\frac{kT}{h}n_{0}e^{-\Delta F_{p}^{*}/kT}e^{-\Delta \phi_{p}^{*}/kT}$$ (34)

where **n**_0_ is the number of polymer molecules per unit volume of solution and $\Delta F_{p}^{*}$ is the free energy of activation for a polymer molecule forming an additional step element. The approximate temperature dependence of $\Delta \phi_{p}^{*}$ can be obtained from eqs (28) and (31):

$$\Delta \phi_{p}^{*}=\frac{2C^{2}\sigma_{s}^{2}\sigma_{e}T_{m}^{2}}{{\left(\Delta h_{f}\right)}^{2}{\left(\Delta T\right)}^{2}}.$$ (35)

Hence,

$$\ln\frac{I}{I_{0}}=-\frac{\Delta H_{p}^{*}}{kT}-\frac{\alpha }{T^{2}{\left(\Delta T\right)}^{2}},$$ (36)

where $I_{0}=\left(kT/h\right)n_{0}\;\exp\;\left(\Delta S_{p}^{*}/k\right)$, and $\alpha =2C^{2}\sigma_{s}^{2}\sigma_{e}T_{m}^{2}/{\left(\Delta h_{f}\right)}^{2}k$. (Here we have set $\Delta F_{p}^{*}=\Delta H_{p}^{*}-T\Delta S_{p}^{*}$) Equation (36) is seen to be of the same form as eq (18) except for the relatively unimportant factor $T_{m}^{2}/T^{2}$. Thus the temperature dependence of the nucleation rate at moderate supercooling is predicted to be similar to that of bundlelike nuclei in a bulk polymer in region *A*.

At this point it is convenient to indicate qualitatively why the nucleus with chain folds described by eq (29–31) and eq (34–36) is the most probable in dilute solution. The basic reason for this behavior is as follows: The free energy required to form a critical bundlelike nucleus in a very dilute solution is greater than the free energy required to form a critical nucleus with loops. This happens because the selection of segments to form the bundlelike crystals requires *many* polymer molecules to be gathered together. This leads to an important change in the difference between the configurational entropy of the crystalline state and the solution state. The change in entropy increases the free energy required to form a critical bundlelike nucleus. This effect is absent or greatly reduced for nuclei with loops, since such nuclei can be formed with a *single* polymer molecule, or a very few polymer molecules. Then stable nuclei with loops are formed much more rapidly than stable bundlelike nuclei from a sufficiently dilute solution. To be more quantitative, it will be shown in section 3.2 that when polyethylene is dissolved in xylene, crystallization will proceed primarily by formation of stable nuclei with loops when $v_{2}\bar{<}0.001$, where *v*_2_ is the volume fraction of polymer. It should be pointed out that while diffusional effects in dilute solutions will tend to reduce the rate of formation of bundlelike nuclei even further, these effects are important only at very low concentrations, where the reduction in configurational entropy has already effectively eliminated the formation of bundlelike nuclei.

The above arguments, concerning the entropy contribution to the free energy required to form a critical nucleus from a dilute solution, also apply to the entropy contribution to the free energy of a grown crystal. It will be shown that a loop-type crystal is more stable than a bundlelike crystal of the same shape and volume in a sufficiently dilute solution.

Brief consideration will now be given to certain aspects of the overall kinetics of crystallization. When a stable nucleus is formed, the nucleus will continue to grow until the molecule is consumed, forming a primary crystallite. At exceedingly low concentrations, where the polymer molecules are very widely separated, and long-range diffusion important, it is possible that the crystallization might proceed mainly through formation of such primary crystallites.

Since the birth time of such a crystallite is essentially the time required to form the critical nucleus, the time required for complete growth being negligible in comparison, the process will in effect be equivalent to sporadic formation of objects (primary crystallites) that do not grow. In this case, *n* would be unity in the *free* growth rate expression

$$\chi^{\prime }=Z_{n}\;t^{n}.$$ (37)

(Note that *n* = 1 in this case is not to be interpreted in the customary manner as one-dimensional growth of objects born at *t* = 0.) At more moderate concentrations, where the degree of crystallinity could be more readily measured, subsequent growth of each nucleus would proceed through secondary nucleation of other adjacent polymer molecules. This nucleation will occur principally on the lateral surfaces of the growing crystal, leading to growth of the **x** and **y** dimensions, because the energy of formation is much smaller for nucleation on the lateral surfaces than on the end surfaces, which contain the folds. The relationship between χ*′*, and the actual mass fraction of polymer crystallized will be given in section 3.3. Then we expect the nuclei, which are born sporadically in time, to grow principally in a two-dimensional manner leading to an overall crystallization isotherm described by *n* = 3. As the crystallization proceeds, *n* will drop in value due to diffusional effects and the consumption of polymeric material. The secondary nucleation mechanism will be discussed further in section 3.3.

### 3.2. Detailed Analysis of Homogeneous Nucleation Rate and Constancy of Step Height in the Primary Crystallite

In section 3.1. we have outlined in simplified form the principal features of homongeneous nucleation from dilute solution. In the present section we shall treat the nucleation process in greater detail with particular emphasis on the variation in step height of the nuclei. We shall at first discuss an ensemble of nuclei, each of which is characterized by a fixed step height **l**, where **l** may differ from $l_{p}^{*}$. The objective is to calculate the distribution in step height in the stable nuclei formed in such a system. Later the assumption that each nucleus in the set has a fixed step height will be relaxed, and found not to alter the general findings (see also appendix 5.1.). In this calculation the edge energy ***ϵ****_p_* will be equated to zero. Its inclusion would not alter the results in an important manner, but would needlessly complicate the analysis at this stage.

Consider a primary nucleus that is composed of ***v*** step elements, all of length **l**. The energy of such a nucleus was given in eq (22) and is rewritten here with ***ϵ****_p_*=0:

$$\Delta \phi_{p}=2va\sigma_{e}+Cl\sqrt{va}\;\sigma_{s}-val\Delta f.$$ (38)

The energy surface represented by this equation is plotted in figure 6. Under the present assumption, a nucleus of ***v*** step elements of length **l** can change by an elementary process only to nuclei of either ***v***−1 or ***v***+1 step elements of length **l**. A stable nucleus of length **l** must be formed through the progressive addition of step elements until the free energy, **Δ*ϕ****_p_*, is negative. Then the path of nucleation will be along the points

$$v_{m},v_{m}+1,v_{m}+2\ldots .,$$ (39)

where ***v****_m_* is the minimum size of a nucleus. Two such paths are shown in figure 6, and will be discussed in more detail shortly

It should be noted that if an embryo is to become stable, it must possess a length, **l**, greater than a certain minimum. This can be seen clearly if eq (38) is differentiated with respect to ***v***:

$$\frac{\partial \Delta \phi_{p}}{\partial v}=\frac{C\sigma_{s}\sqrt{a}l}{2\sqrt{v}}+a(2\sigma_{e}-l\Delta f).$$ (40)

It can be seen by inspection that the right hand side of eq (40) decreases monotonically with increasing ***v*** for all positive ***v***. If the length of the embryo is so small that **l**<2***σ****_e_*/**Δf**, then **∂Δ*ϕ****_p_*/**∂*v*** is always positive. The free energy of such embryos will increase indefinitely with the addition of step elements, and the embryos can never become stable. Then stable nuclei can be formed only when

$$l>\frac{2\sigma_{e}}{\Delta f}=\frac{1}{2}l_{p}^{*}.$$ (41)

When eq (41) is satisfied, **∂Δ*ϕ****_p_/***∂*v*** decreases monotonically with ***v*** from a positive value to a finite negative value. Under these circumstances the free energy of the embryos, **Δ*ϕ****_p_*, increases with the addition of step elements until a maximum value, $\Delta \phi_{p}^{‡}$, is reached when there arc ***v***^‡^ step elements. The free energy decreases monotonically as further step elements are added. Two such paths of nucleation are shown in figure 6.

The number of step elements in the embryo at the energy barrier can be calculated by equating **∂Δ*ϕ****_p_*/**∂*v*** to zero in eq (40):

$$v^{‡}=\frac{{\left(C\sigma_{s}l\right)}^{2}}{4a{\left(l\Delta f-2\sigma_{e}\right)}^{2}}.$$ (42)

The energy barrier is

$$\Delta \phi_{p}^{‡}=\frac{{\left(C\sigma_{s}l\right)}^{2}}{4(l\Delta f-2\sigma_{e})}.$$ (43)

We have already seen that this energy barrier is a minimum when

$$l_{p}^{*}=\frac{4\sigma_{e}}{\Delta f},$$ (44)

and that this minimum energy barrier is

$$\Delta \phi_{p}^{*}=\frac{2C^{2}\sigma_{s}{\;}^{2}\sigma_{e}}{{\left(\Delta f\right)}^{2}}.$$ (45)

With some algebraic manipulation of eqs (43), (44) and (45), we may write the energy barrier, $\Delta \phi_{p}^{‡}$, as

$$\Delta \phi_{p}^{‡}=\Delta \phi_{p}^{*}\left[1+\frac{{\left(l/l_{p}^{*}-1\right)}^{2}}{1+2(l/l_{p}^{*}-1)}\right]$$ (46)

This expression gives the value of the barrier hindering the formation of a stable nucleus composed of step elements of length **l**; these values of $\Delta \phi_{p}^{‡}$, lie on the ridge D–E on the free energy surface shown in figure 6. Since the energy barrier to be surmounted is a minimum at $l=l_{p}^{*}$, where there is a saddle point in the ridge, it can be seen intuitively that the rate of formation of nuclei will be largest when the nuclei have lengths near this value.

The effect of deviations of length from $l_{p}^{*}$ on the rate will now be established. In appendix 5.1., the rate of formation of these nuclei was calculated using the procedure of Turnbull and Fisher [8]. It was determined that the number of stable nuclei with lengths between **l** and **l**+*d***l** formed per unit time per unit volume of solution is

$$i(l)dl=\xi dl\frac{n_{0}kT}{h}e^{-\Delta F_{p}^{*}/kT}e^{-\Delta \phi_{p}^{‡}/kT}$$ (47)

where

$$\xi =\frac{a^{2}\sqrt{2\sigma_{e}}}{l}\frac{{\left(l\Delta f-2\sigma_{e}\right)}^{\frac{3}{2}}}{\pi {\left(kT\right)}^{2}}.$$

The value of *ξ* may be considered valid at best to within an order of magnitude. However, it varies slowly with l compared to the factor $\exp\;[-\Delta \phi_{p}^{‡}/kT]$. Substitution of eq (46) into eq (47) leads to

$$\begin{array}{l} i(l)=\xi \frac{n_{0}kT}{h}\exp\;\left[-\frac{\Delta F_{p}^{*}}{kT}-\frac{\Delta \phi_{p}^{*}}{kT}\right]\\ \;\times \exp\;\left\{-\frac{\Delta \phi_{p}^{*}}{kT}\cdot \left[\frac{{\left(l/l_{p}^{*}-1\right)}^{2}}{1+2(l/l_{p}^{*}-1)}\right]\right\}. \end{array}$$ (48)

This equation shows clearly that the nucleation rate is most rapid when $l=l_{p}^{*}$, and that the distribution in the lengths of the nuclei formed will become sharper as the height of the barrier at the saddle point, $\Delta \phi_{p}^{*}$, increases.

The derivation given above can be generalized further to include various step heights **l_1_**, **l**_2_…, for each individual nucleus, rather than just one. The general conclusion is that eq (48) is a reasonable measure of the variation in length of the stable nuclei formed. The fraction of stable nuclei with lengths between **l**_1_ and **l**_2_ can be calculated directly from eq (48):

$$\begin{array}{l} f(l_{2},l_{1})=\frac{\int_{l_{1}}^{l_{2}}i(l)dl}{\int_{l_{p/2}^{*}}^{\infty }i(l)dl}\\ \;=\frac{\int_{l_{1}}^{l_{2}}\exp\;\left\{-\frac{\Delta \phi_{p}^{*}}{kT}\left[\frac{{\left(l/l_{p}^{*}-1\right)}^{2}}{1+2(l/l_{p}^{*}-1)}\right]\right\}dl}{\int_{l_{p/2}^{*}}^{\infty }\exp\;\left\{-\frac{\Delta \phi_{p}^{*}}{kT}\left[\frac{{\left(l/l_{p}^{*}-1\right)}^{2}}{1+2(l/l_{p}^{*}-1)}\right]\right\}dl} \end{array}$$ (49)

The lower limit of the integral in the denominator represents the smallest possible size of a stable nucleus. This expression will prove useful in estimating the percentage variation of **l** about its probable value, $l_{p}^{*}$.

The total nucleation rate is obtained by integration of eq (48). When $(\Delta \phi_{p}^{*}/kT)>>1$, the number of stable nuclei formed per unit time per unit volume of solution is

$$I=\int_{l_{p/2}^{*}}^{\infty }i(l)dl=K\frac{n_{0}kT}{h}e^{-\Delta F_{p}^{*}/kT}e^{-\Delta \phi_{p}^{*}/kT}$$ (50)

where $K={\left(2\sigma_{e}\right)}^{\frac{3}{2}}(\Delta f)a^{2}/\pi^{\frac{1}{2}}C\sigma_{s}{\left(kT\right)}^{\frac{3}{2}}$. For most cases of interest, *K* is within an order of magnitude of unity, and following Turnbull and Fisher, we shall set *K* equal to unity. Then we have for the nucleation rate

$$I=\frac{n_{0}kT}{h}e^{-\Delta F_{p}^{*}/kT}e^{-\Delta \phi_{p}^{*}/kT}.$$ (51)

Substitution of eqs (45) and (28) into eq (51) yields

$$I=\frac{n_{0}kT}{h}e^{-\Delta F_{p}^{*}/kT}\exp\;\left[-\frac{2C^{2}\sigma_{s}^{2}\sigma_{e}T_{m}^{2}}{kT{\left(\Delta h_{f}\right)}^{2}{\left(\Delta T\right)}^{2}}\right].$$ (52)

(loop nuclei in dilute solution)

At temperatures near **T***_m_*, it is clear that the last factor furnishes the principal temperature dependence of ***I***.

We turn now to some numerical values to illustrate the general characteristics of the nucleation of crystals with loops. Unfortunately, no complete set of experimental data is available, so we must be satisfied with estimates. Attention will be centered on the case of the crystallization of polyethylene from xylene at 90° C, for Keller and O’ Connor [1] have measured the step height as formed under these conditions and found it to be about 140 A. It should be noted that xylene is a reasonably good solvent, so that there is no separation into two liquid phases at low concentrations. This condition must be satisfied for the theory given here to be applicable. An estimate on ***σ****_e_* may be obtained by combining eq (28) and eq (29) so that

$$\sigma_{e}=\frac{1}{4}\Delta h_{f}l_{p}^{*}(\Delta T/T_{m}),$$ (53)

where we have approximated the step height of the crystal by $l_{p}^{*}$ in this equation. Quinn and Mandelkern [14] have measured the heat of fusion of polyethylene and have found it to be 67 cal·g^−1^. From Bunn’s X-ray data [12] on bulk crystalline polyethylene at room temperature, it may be estimated that the cross-sectional area of the chain segment is 18.5×10^−16^ cm^2^, and that the volume of each –CH_2_CH_2_– unit is 47×10^−24^ cm^3^. (These values are adjusted to be correct at 90° C.) It is determined from these results that Δh*_f_*, the heat of fusion per unit volume of crystal, is 2.8×10^9^ erg·cm^−3^. We will (somewhat arbitrarily) assume that this is the heat of fusion **Δh***_f_* in the presence of large quantities of solvent. It is then found that

$$\sigma_{e}=980\;\left[\frac{T_{m}-363}{T_{m}}\right]\;\text{erg}\cdot {\text{cm}}^{-2}.$$ (54)

The melting point of polyethylene crystals in very dilute solutions of xylene is difficult to estimate with confidence. A lower limit on **T***_m_* of 95° C may be calculated from the theory of the depression of the melting point of a polymer by diluent [15] with the interaction parameter χ_1_=0. The true value of **T***_m_* is very probably somewhat higher, since χ_1_ almost certainly differs from zero. It is considered probable that **T***_m_* lies between 95° C and 120° C for this particular solvent. Then ***σ****_e_* lies between 13 and 75 erg·cm^−2^. A value of ***σ****_e_* in this range seems reasonable, since it corresponds to an energy of loop formation of 0.7 to 4 kcal/mole of loops. We should expect polymers with stiffer chains to possess higher values of ***σ****_e_* than polyethylene. In continuing the numerical analysis ***σ****_e_* will be set equal to 30 erg·cm^−2^. In eq (54) this implies **T***_m_* = 374.5° K, and therefore **Δf**=1.17×10^8^ erg-cm^−3^ at 90°C. To estimate the value of $\Delta \phi_{p}^{*}/kT$ at this temperature, the shape constant C must be known. If it is assumed that the shape of the cross section of the nucleus is a parallelogram with an acute angle, *ψ* = 70°, between the sides, *C*=4.13. Then from eq (45) or (31)

$$\frac{\Delta \phi_{p}^{*}}{kT}=1.5\sigma_{s}^{2}$$ (55)

at *T*=90° C. A reasonable value of ***σ****_s_* might lie between 5 and 20 erg·cm^−2^ (Thomas and Stavely [16] have found ***σ***=20.4 erg·cm^−2^ for benzene). Then $\Delta \phi_{p}^{*}/kT$ must lie between 38 and 600.

Crystallization could not be observed if $\Delta \phi_{p}^{*}/kT$ possessed a value of 600. For the particular case of crystallization from a 0.01 percent solution of polyethylene in xylene, $\Delta \phi_{p}^{*}/kT$ must be well below 100. If ***σ****_s_* lies between 5 and 6 erg·cm^−2^, $\Delta \phi_{p}^{*}/kT$ lies between about 40 and 50. In any case it is clear that the value of $\Delta \phi_{p}^{*}/kT$ for slow but measurable crystallization processes is a large number. This is because $\sigma_{s}^{2}\sigma_{e}$ possesses large values for nuclei with folded chains. One might expect that $\sigma_{s}^{2}\sigma_{e}$ lies between 500 and 10,000 erg^3^·cm^−6^ for most polymers where the nuclei involve chain folding. The quantity $\sigma_{s}^{2}\sigma_{e}$ relevant to the case of bundlelike nuclei in bulk is much smaller, values of 25 to 250 erg^3^·cm^−6^ being reasonable.

The distribution in step heights of critical nuclei about $l_{p}^{*}$ can be estimated from eq (49). If when polyethylene is crystallized from a 0.01 percent solution of xylene, $l_{p}^{*}=140$ A and $\Delta \phi_{p}^{*}/kT=50$, the evaluation of eq (49) shows that 73 percent of the critical sized nuclei have step heights between 126 and 157 A. This distribution is sufficiently narrow so that several orders of low angle X-ray scattering might be expected,^6^ if the grown crystals possess this distribution. This is in satisfactory agreement with experiment.

These numerical values will be discussed further after the growth of the crystals through secondary nucleation of other polymer molecules has been investigated in the next section.

We now wish to show in some detail that the formation of bundlelike nuclei in sufficiently dilute solution is negligible compared to nucleation through chain folding. The nucleation rate of bundlelike nuclei of circular cross section in the presence of diluent, *I_d_*, has been calculated by Mandelkern [13]. With appropriate changes in notation his result is

$$I_{d}=I_{od}e^{-\Delta F_{d}^{*}/kT}\exp\;\left[-\frac{8\pi \sigma_{sd}^{2}\sigma_{ed}}{kT{\left(\Delta f_{d}\right)}^{2}}\right]\;\exp\;\left[\frac{4\pi \sigma_{sd}^{2}}{a{\left(\Delta f_{d}\right)}^{2}}\log_{e}v_{2}\right]$$ (56)

(bundlelike nuclei in dilute solution)

where $\Delta F_{d}^{*}$ is the activation energy required for transport across the nucleus-liquid interface, Δ*f_d_* is the bulk free energy difference per unit volume of crystal between the crystalline and solution states, *σ_sd_* is the free energy required to build a unit area of the lateral surface from the bundlelike crystal in solution, and *v*_2_ is the volume fraction of the polymer in the solution. *σ_ed_* is a surface free energy corresponding to *σ_e_*, the free energy required to build a unit area of end surface in the bulk polymer. *σ_ed_* must be less than ***σ****_e_*, the energy required to form a unit area of a surface containing loops. The pre-exponential factor, *I_od_*, is not particularly sensitive to *v*_2_, varying approximately as the first power of **n**_0_. It is to be expected that *σ_sd_*, Δ*f_d_*, and *a* should have values very nearly equal to ***σ****_s_*, **Δf**, and **a**, the corresponding terms for nuclei with folds. Then the important dependence of *I_d_* upon *v*_2_ occurs in the last factor in eq (56). The last factor decreases very rapidly with decreasing *v_2_*, and the nucleation rate for bundlelike nuclei is reduced correspondingly. Thus at sufficient dilution the nuclei with folds are the preferred type, as may be seen by comparing eq (52) with eq (56).

A more quantitative comparison can be made if eq (52) is divided by eq (56):

$$\frac{I}{I_{d}}=C_{0}\;\exp\;\left\{-\frac{\Delta \phi_{p}^{*}}{kT}\left[1-\frac{4\pi \sigma_{ed}}{C^{2}\sigma_{e}}+\frac{2\pi kT}{C^{2}a\sigma_{e}}\log_{e}\;v_{2}\right]\right\}$$ (57)

where $C_{0}=\left(n_{0}kT/hI_{0d}\right)\;\exp\;[-(\Delta F_{p}^{*}-\Delta F_{d}^{*})/kT]$. In eq (57) it has been assumed that **Δf** = Δ*f_d_*, ***σ****_s_*=*σ_sd,_* and **a**=*a*. Here $\Delta \phi_{p}^{*}$ is the energy required to form a critical nucleus with loops. When ***I***/*I_d_* ≫ 1, the stable nuclei formed are primarily those with loops. It is instructive to evaluate eq (57) for polyethylene crystallized from xylene. *C*_0_ is not very sensitive to either concentration or temperature and probably has a value between 10^−10^ and 1. We find 2*πkT*/*C*^2^**a*σ****_e_* ≅ 1/3, if *≅_e_* is assigned the value of 30 erg·cm^−2^. Then eq (57) becomes

$$\left(I/I_{d})=C_{0}\exp\{-(\Delta \phi_{p}^{*}/kT)[1+\frac{1}{3}\log_{e}v_{2}-(4\pi \sigma_{ed}/C^{2}\sigma_{e})]\right\}$$ (58)

It has been shown that $\Delta \phi_{p}^{*}/kT$ is a large number. If *v*_2_=0.001 and *C*_0_=10^−10^, ***I***/*I_d_* lies between 10^12^ and 10^35^ as $\Delta \phi_{p}^{*}/kT$ varies between 40 and 80. Thus in a 0.1 percent solution of polyethylene in xylene, crystallization should definitely occur through the formation of nuclei with loops. At concentrations near 10 percent ***I***/*I_d_*≪1, and bundlelike nuclei will dominate the crystallization process. The transition between the two types of crystals occurs near *v*_2_=0.01. Equations (57) and (58) must be applied with caution near this transition region for two reasons: (a) the nucleation rate for crystallites with loops was derived for very dilute solutions and is probably inaccurate at higher concentrations; (b) ***I*** and *I_d_* are rates for extreme types of nucleation, and in the transition region the stable nuclei formed are probably partially bundlelike and partially formed through chain folding. Nevertheless eq (57) indicates that there is a fairly sharp value of the volume fraction of polymer, *v*_2(_*_c)_*, such that when *v_2_*>*v*_2(_*_c_*_)_ bundlelike nuclei are formed, and when *v_2_*<*v*_2(_*_c_*_)_ nuclei with loops are formed. If there is some restraint on the radial growth of bundlelike nuclei, such as the type of strain mentioned earlier, stable bundlelike nuclei may be even more difficult to form than has been indicated, and *v*_2(_*_c_*_)_ would have a higher value than that deduced from eq (58). Even without this, the important point remains that looptype nuclei will predominate at low concentration.

In extremely dilute solution the preponderance of nuclei with loops over those that are bundlelike is enhanced even further by diffusional effects. Since at higher concentrations loop nuclei are already the most important in the system, we see no compelling need to give a detailed analysis of the effect of long range diffusion.

The above comparison naturally raises the question of why the configurational entropy contribution to the free energy of formation of a bundlelike nucleus is so much more sensitive to the concentration of the solution than is the corresponding term for a nucleus with folds. Qualitatively this can be answered as follows. In forming a critical bundlelike nucleus the segments of many molecules must be brought together. The entropy reduction in bringing together different polymer molecules in dilute solution is sensitively dependent upon the concentration. In forming a critical nucleus with folds from a single polymer molecule, the segments of this molecule must be brought together in an appropriate manner. There is a corresponding entropy contribution but this contribution does not depend upon the concentration of the solution. This qualitative explanation can be placed on a quantitative basis if a lattice model is used. The lattice model is not accurate for dilute solutions, but calculations based upon it should be roughly correct. It is found that the reduction in entropy due to the gathering of molecules in a bundlelike crystal is – *k* ln *v_2_ per segment* in the cross section of the crystal. This result yields an end surface energy of the form *σ_ed_*–(*kT*/*2a*) log*_e_ v*_2_ and leads to eq (56). By analogy, for a crystal with folds, the reduction in entropy is –*k* ln *v_2_ per polymer molecule* contained in the nucleus. If a single polymer molecule is involved in the formation of a critical nucleus with folds this contribution need not be considered and eq (52) results. If many molecules are involved in the crystal the free energy contribution per unit area of surface of the crystal is −(**l**/*L*)(*kT*/2**a**)log*_e_v_2_*, where **l** is the step height of the crystal, and *L* is the mean length of the polymer molecules. This term is unimportant for high molecular weight polymers. In any case since this “surface energy” term is proportional to the step height of the crystal, it will be included in the bulk free energy difference per unit volume of crystal, **Δf**.

It has already been shown that for kinetic reasons almost all of the critical nuclei possess lengths very close to $l_{p}^{*}=4\sigma_{e}/\Delta f$. The critical nucleus can often be formed from a single polymer molecule. After these nuclei are formed, the remainder of the polymer molecule forming the nucleus will “crystallize” onto the nucleus until a primary crystallite is formed by a single molecule, which has a crystalline volume **a***L* where *L* is the length of the molecule. The distribution in step heights of this primary crystallite will now be briefly considered.

**Figure 7:**
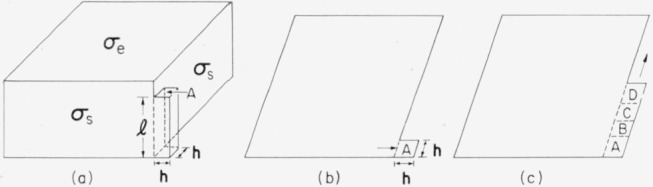
The addition of step elements around a “corner” of a nucleus. (a) and (b): Perspective and a top view of the addition of a step element. *A*, to a nucleus. The step element *A* must be added in order that a monomolecular layer can be established along the face of the crystal, (c) Top view of the step elements *B, C, D,…*, that can be added to the crystal face after the first step element, *A*, has attached to the “corner” of the nucleus.

It will be assumed that the primary crystallite will be formed from the critical nucleus by the addition of step elements in the manner shown by figure 5a, so that the step elements are added in a monomolecular layer to the existing already “crystallized” nucleus. This monomolecular layer will be added to one side of the nucleus until a “corner” of the nucleus is reached. At this stage the step height may be maintained near 4***σ****_e_*/**Δf** although lower values may be attained. When the monomolecular layer of step elements reaches the “corner” of the nucleus, the next step element must be added so that it extends beyond the corner of the nucleus. This situation is shown schematically in figure 7a and 7b, where the additional step element is designated by *A*.

A monomolecular layer may then be added along the surface of the nucleus by the addition of step elements *B*, *C*, *D*, etc., as is shown in figure 7c. The calculation of the rate at which this monomolecular layer is deposited on the surface of the nucleus is complicated by two factors: (a) an accurate expression for the free energy of such a monomolecular layer is lacking, and; (b) the fundamental expression for the rate of crystallization of a monomolecular layer is somewhat different from the expression used for the primary crystallization.^7^ The first complication will be avoided by using a purely geometric model for the free energy of the monomolecular layer. Thus each step element will be assumed to be a parallelepiped which has the surface energies appropriate to the bulk crystal. This model should yield answers that are qualitatively correct. Then if the step height of the added step elements *A, B, C, D,…*, is less than the step height of the nucleus, the free energy required to add ***v*** step elements around the corner of the nucleus is

$$\Delta \phi^{\prime }=2h\sigma_{s}l+2va\sigma_{e}-val\Delta f$$ (59)

where $h={\left(a/\sin\psi \right)}^{\frac{1}{2}}$ is the length of the side of the end surface of the step element. The usual method of finding the activation energy by setting **∂Δ*ϕ***/**∂l**=0, **∂Δ*ϕ***/**∂*v*a**=0, and substituting into **Δ*ϕ***, is inapplicable in the case of monomolecular growth with ***v***=1, since the free energy surface does not have a saddle point that corresponds to a minimum activation energy. Hence we must examine the free energy ridge for ***v***=1 over which the system must pass in more detail.

The increment of energy required to add the step element at the corner of the nucleus is obtained by setting ***v***=1 in eq (59):

$$\Delta {\phi^{\prime }}^{‡}=2h\sigma_{s}l-a(l\Delta f-2\sigma_{e}).$$ (60)

At the degree of supercooling in the range of experimental interest, 2**h*σ****_s_*>**aΔf**, and therefore **Δ*ϕ***′^‡^ increases with increasing **l**. Of course, if the length of the step element becomes larger than the nucleus, additional terms increase **Δ*ϕ***′^‡^ even more rapidly with **l**. The addition of further step elements *B*, *C, D,…*, change the free energy by a constant amount

$$\Delta \phi_{v+1}^{\prime }-\Delta \phi_{v}^{\prime }=-E=-a(l\Delta f-2\sigma_{e}).$$ (61)

It is clear that the step element must have a length greater than 2***σ****_e_*/**Δf** or the resulting crystal is unstable.

The addition of the corner step element *A* requires an activation barrier **Δ*ϕ***′^‡^. Addition of further step elements of this length reduces the free energy by an amount *E* per step element. In appendix 5.2 it is shown that the equilibrium rate of deposition of monomolecular layers of step heights between **l** and **l** + *d***l** is

$$\begin{array}{l} rdl=dl\frac{C_{1}NkT}{h}e^{-\Delta F^{*}/kT}e^{-\Delta {\phi^{\prime }}^{‡}/kT}\\ \;\cdot \left\{\frac{2\sinh(E/2kT)}{1+2e^{-\Delta {\phi^{\prime }}^{‡}/2kT}\;\sinh\;(E/2kT)}\right\} \end{array}$$ (62)

where ΔF* is the activation energy of the elementary process of adding the step element, **N** is the number of primary nuclei which are growing, and *C*_1_ is a normalization constant.

The rate of deposition of the monomolecular layer depends upon the step height, **l**, of the layer. At **l**=2***σ****_e_*/**Δf** this rate is zero and as **l** increases the rate increases until a maximum is attained and then the rate decreases with a further increase of **l**. It will be shown that the rate is appreciable in only a narrow range of values of **l**.

A numerical analysis shows that for the case in which we are interested, the rate in eq (62) can be approximated by

$$\begin{array}{l} r=2\frac{kT}{h}NC_{1}{\;}^{-\Delta F^{*}/kT}e^{-\Delta {\phi^{\prime }}^{‡}/kT}\sinh\;\left[\frac{E}{2kT}\right]\\ \;=2\frac{kT}{h}NC_{1}e^{-\Delta F^{*}/kT}e^{-I(2h\sigma_{s}-a\Delta f)/kT}e^{-2a\sigma_{e}/kT}\\ \;\sinh\;\left[\frac{a(l\Delta f-2\sigma_{e})}{2kT}\right] \end{array}$$ (63)

The mean length of the step height of this layer can be taken as

$$\bar{l}=\frac{\int_{2\sigma_{e}/\Delta f}^{\infty }\mathrm{lr}\;dl}{\int_{2\sigma_{e}/\Delta f}^{\infty }r\;dl}=\frac{2\sigma_{e}}{\Delta f}+\left[\frac{2kT}{4h\sigma_{s}-3a\Delta f}+\frac{2kT}{4h\sigma_{s}-a\Delta f}\right].$$ (64)

Similarly, the mean square deviation is

$$\begin{array}{l} <{\left(l-\bar{l}\right)}^{2}>=\frac{\int_{2\sigma_{e}/\Delta f}^{\infty }{\left(l-\bar{l}\right)}^{2}r\;dl}{\int_{2\sigma_{e}/\Delta f}^{\infty }r\;dl}\\ \;=\left\{\frac{{\left(2kT\right)}^{2}}{{\left(4h\sigma_{s}-3a\Delta f\right)}^{2}}+\frac{{\left(2kT\right)}^{2}}{{\left(4h\sigma_{s}-a\Delta f\right)}^{2}}\right\}. \end{array}$$ (65)

The square root of eq (65) can be used as a measure of the deviation of the step heights from the mean value, $\bar{l}$.

When **h*σ****_s_*>>**aΔf**, eqs (64) and (65) become

$$\bar{l}=\frac{2\sigma_{e}}{\Delta f}+\frac{kT}{h\sigma_{s}}$$ (66a)

and

$$<{\left(l-\bar{l}\right)}^{2}>=\frac{1}{2}{\left(\frac{kT}{h\sigma_{s}}\right)}^{2}.$$ (66b)

Now if **h**=4.2 A, ***σ****_s_*= 6 erg·cm^−2^, and *T*=363° K, then *kT*/**h*σ****_s_* is about 20 A. The mean deviation about this average value is about 14A. Then when the monomolecular layer passes around the “corner” the length of the step height falls from 4***σ****_e_*/**Δf** to a value slightly greater than 2***σ****_e_*/**Δ**f. The distribution of step heights about this mean value is quite sharp. Every time the monomolecular layer reaches a “corner” this identical situation will be repeated. It might be expected that there is a tendency for the step height to increase, as the monomolecular layer is being crystallized along the side of a primary nucleus. An analysis of this process shows that the step height will remain near that given by eq (66a).

In summary, it can be said that if the edge energy ***ϵ****_p_* is negligible, the primary crystallite will have an interior section which has a step height $l_{p}^{*}=4\sigma_{e}/\Delta f$, and the outer section will have a step height near $\bar{l}=2\sigma_{e}/\Delta f+kT/h\sigma_{s}$. More will be said of this process in the next section. If the edge energy ***ϵ****_p_* is not negligible it will affect the growth of the critical nucelus into a primary crystallite. This effect will also be discussed in the next section.

### 3.3. Constancy of Step Height in Overall Growth Process and Volume Increase of a Folded Crystal Dilute Solution

When a primary crystal has been formed, it can grow by the addition of other polymer molecules upon it, one by one. This crystallization will proceed by the formation of a secondary nucleus by a single molecule upon the lateral surface of the crystal. This growth of the crystals is treated in this section with emphasis on two points. First we wish to demonstrate that the step height of the growing crystal has a tendency to remain at a constant value l* for kinetic reasons. Second, it is desired to obtain appropriate expressions describing the volume rate of growth of these crystals.

Before we discuss growth through secondary nucleation on the lateral surfaces, our neglect of nucleation of the end surfaces must be justified. The end surface of the primary crystal is composed of loops formed by the folding of polymer molecules. The end surface of a secondary nucleus is also composed of folds. Thus there is a distinct boundary between the crystal and such a secondary nucleus. The effect of any affinity between the loops in the two end surfaces upon the free energy required to form a secondary nucleus is probably small. Then the free energy required to form a secondary nucleus upon the end surface of the crystal is almost as great as that required to form a primary nucleus. Some growth on the end surface will, of course, occur. However, by the arguments given above, the step height will be practically identical to that of the primary crystallite. Secondary growth of this type can lead to small patches of secondary growth on the primary crystallite, or in other cases to a distinct pyramidal appearance due to successive layers being formed. These effects should be subdued by forming crystals at very low concentration.

The free energy required to form a secondary nucleus upon the lateral surface of the crystal is considerably smaller than that required to form a primary nucleus. The volume growth of a crystal proceeds through the formation of a stable secondary nucleus on the growing (lateral) surface of the crystal followed by complete “crystallization” of the entire new molecule. The rate of addition of molecules to the crystal will be the average number of molecules in contact with the growing surface times the rate at which one of these molecules forms a stable nucleus, **p***_g_*. The quantity, **p***_g_* can be calculated by the method of Turnbull and Fisher [8].

$$p_{g}=\frac{kT}{h}e^{-\Delta F^{*}/kT}e^{-\Delta \phi_{g}^{*}/kT}$$ (67)

where $\Delta \phi_{g}^{*}$ is the free energy required to form a secondary or growth nucleus of critical size. The process of the formation of a fold by a molecule should be the same in primary and secondary nuclei, so that we anticipate $\Delta F_{g}^{*}=\Delta F_{p}^{*}$. This expression holds for growth where the activated state is reached through many successive elementary processes. Later, the case of growth through addition of a monomolecular layer will be considered, and eq (67) will be modified accordingly.

The free energy of secondary nuclei of critical size, $\Delta \phi_{g}^{*}$, will be considerably smaller than the corresponding energy for primary nuclei. The calculation of $\Delta \phi_{g}^{*}$ requires an accurate expression for the free energy of a secondary nucleus, $\Delta \phi_{g}$. We can obtain such an expression when the shape of the secondary nucleus is known and the number of segments in a cross-sectional area is large. It is probable that neither condition is satisfied for the secondary nucleus. We will, however, consider two extreme cases: (1) the cross section of the secondary nucleus has the same shape as that of the primary nucleus, and, (2) the secondary nucleus consists of a single layer of enfolded sections of a polymer molecule upon the surface of the crystal.

**Figure 8:**
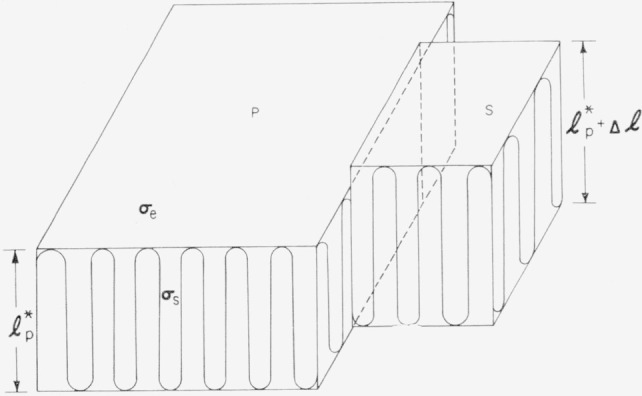
Schematic diagram of primary crystal, P, with a hypothetical secondary nucleus of the same cross-sectional shape, S, upon lateral surface of the crystal. A secondary nucleus or embryo where **Δl**≠0 is shown in the text to be considerably less stable than one of length $l_{p}^{*}$.

The former case where the shape of the cross section of the secondary and primary nuclei are the same is not likely to be correct, but it has the advantage that an accurate expression for its free energy may be written down explicitly. In figure 8, a secondary nucleus of this type is shown on the lateral surface of the larger primary crystal. The free energy required to form this nucleus is the difference between the free energy required to form the total crystalline region *P+S* shown in figure 8, and the free energy required to form the crystal, *P.* If there are ***v*** step elements in the secondary crystal, the free energy required to form the secondary nucleus is

$$\Delta \phi_{g}^{\prime }=2va\sigma_{e}+\frac{C}{2}\sqrt{va}\sigma_{s}l_{p}^{*}-val_{p}^{*}\Delta f.$$ (68)

The equation above applies to the case where both the crystal and the secondary nucleus possess the critical length $l_{p}^{*}$. It should be noted that the secondary nucleus possesses only one-half as much lateral surface energy as a primary nucleus of the same size and shape. We easily find that

$$\Delta \phi_{g}^{*}=\frac{\Delta \phi_{p}^{*}}{4}.$$ (69)

Then the activation barrier of a secondary nucleus would be only one-quarter of that required to form a primary nucleus. If the height of the step elements in the secondary nucleus is allowed to be $l_{p}^{*}+\Delta l$, the free energy required to form such a secondary nucleus can be calculated by the usual methods. It is found that the energy barrier $\Delta \phi_{g}^{\prime ‡}$ which such a nucleus must surmount is

$$\begin{array}{c} \Delta \phi_{g}^{\prime ‡}=\frac{1}{4}\Delta \phi_{p}^{*}+\frac{\Delta \phi_{p}^{*}}{2}\frac{\left(\Delta l\right)}{l_{p}^{*}}\text{for}\;\Delta l\geq 0,\\ \Delta \phi_{g}^{\prime ‡}=\frac{1}{4}\Delta \phi_{p}^{*}+\frac{\Delta \phi_{p}^{*}}{4}\frac{{\left(\Delta l\right)}^{2}}{l_{p}^{*}\left(l_{p}^{*}+2\Delta l\right)}\text{for}\;\Delta l\leq 0. \end{array}$$ (70)

The variation in lengths of secondary nuclei will thus be small, since we have seen that $\Delta \phi_{p}^{*}$ is large.

If the primary nucleus has a length **l** which is greater than $l_{p}^{*}$, the secondary nucleus will possess a length very close to $l_{p}^{*}$. It is clear that if the secondary nucleus has the same cross-sectional shape as the primary nucleus, the step height of the crystal will not increase as the latter body grows.

The activation barrier required to form a critical secondary nucleus of the same shape as the primary nucleus is large. It is therefore probable that the secondary nucleus of critical size is a *monomolecular* layer of step elements that lie along the growing crystal face. An accurate expression for the free energy of such a nucleus is not available, but the same assumptions that were used in the previous section may be applied here. The free energy required to nucleate on the growing crystal face is the same as that required for a monomolecular layer to turn a corner and grow on a new crystal face. Thus **Δ*ϕ****_g_* is identical to **Δ*ϕ***′ in eq (59). From the results of the previous section concerning the formation of a primary crystallite it can be concluded that if the edge energy, ***ϵ***, is negligible the crystal will grow with a constant step height, l*, which is given approximately by

$$l*=\frac{2\sigma_{e}}{\Delta f}+\frac{kT}{h\sigma_{s}}.$$ (71)

When the monomolecular layers have completely encircled the growing crystallite, it is improbable that additional layers will have step heights appreciably larger than l* since such layers would extend above the growing crystal face and therefore would require more free energy to construct. Thus the distribution will be somewhat sharper than that implied by eq (65), and the step height may decrease slightly from the value given by eq (71). In any case the crystal will grow with a very narrow distribution of step heights about l*, and the variation of step height should be approximately 1/2^½^(*kT*/**h*σ****_s_*).

For this type of secondary nucleus, **p***_g_* is obtained by the integration of (**r**/**N**)*d***l** over all permissible values of **l**. With a suitable choice of *C*_1_ in eq (63) we have approximately

$$p_{g}=\frac{kT}{h}\left(\frac{a\Delta f}{2h\sigma_{s}}\right)\;\exp\;\left(-\frac{\Delta F_{g}^{*}}{kT}\right)\;\exp\;\left\{-\frac{4h\sigma_{s}\sigma_{e}}{kT\Delta f}\right\}.$$ (72)

In this case log **p***_g_* varies approximately as (**ΔT**)^−1^ for moderate supercooling.

Price [19] has independently considered the growth of crystals with folds through nucleation of mono-molecular layers.

At this point it is appropriate to discuss the possibility of an edge free energy affecting the growth process appreciably. An edge free energy in a monomolecular secondary nucleus can be considered to arise as follows. If the growing crystal has flat surfaces containing loops, the packing of the loops increases the stability of the crystal. If a monomolecular layer is placed upon the growing surface of the crystal, where the step height of the growing surface differs from that of the layer, the loops in the monomolecular layer cannot be as efficiently packed as if they coincided with the flat surfaces of the crystal. This will lead to an edge energy appearing in the expression for the free energy required to form this monomolecular layer

$$\Delta \phi_{g}^{\prime \prime }=2h\sigma_{s}l+2va\sigma_{e}+vh\epsilon -val\Delta f$$ (73)

where ***ϵ*** is the free energy required to form a unit length of “edge” in the monomolecular layer. The introduction of the parameter ***ϵ*** in eq (73) will not affect the general conclusions previously obtained, but will affect the quantitative results.

We are justified in considering this case since it will he shown in section 3.5. that it can be experimentally determined whether ***ϵ*** is negligible or not. Equation (73) applies to a layer where the step height of the monomolecular layer is less than that of the growing crystal surface.

The free energy required to form a monomolecular layer with a larger step height than the growing crystal surface requires the addition of a term 2***v*h*σ****_s_***Δl** to eq (73), where **Δl** is the difference in step height. The free energy required to form a monomolecular layer with the same step height as the growing crystal surface is

$$\Delta \phi_{g}^{\prime \prime \prime }=2h\sigma_{s}l+2va\sigma_{e}-val\Delta f,$$ (74)

i.e. no term in ***ϵ*** appears.

Inspection of eq (73) shows that when **l**<2***σ****_e_****/*Δf**+ **h*ϵ*/aΔf**, $\Delta \phi_{g}^{\prime \prime }$ increases with increasing ***v***. This will hold true until the monomolecular layer extends around the entire crystal when a maximum free energy will be attained. Further additions of step elements would then reduce the free energy. The activation barrier would be very large particularly if the crystal were large. The formation of a stable nucleus with a step height less than 2***σ****_e_****/*Δf**+ **h*ϵ*/aΔf** would undoubtedly proceed through the formation of a differently shaped nucleus, but would in any case require a very large activation barrier. We see therefore that we need only consider the case

$$l>\frac{2\sigma_{e}}{\Delta f}+\frac{h\epsilon }{a\Delta f}.$$ (75)

Inspection of eq (73) shows that it is identical to eq (59) if ***σ****_e_* in the former equation is replaced by (***σ****_e_+***h*ϵ***/2**a**) in the latter. It is therefore unnecessary to repeat the calculations, and the step height of the monomoleuclar layer will be

$$l*=\frac{2\sigma_{e}}{\Delta f}+\frac{h\epsilon }{a\Delta f}+\frac{kT}{h\sigma_{s}}.$$ (76)

After the step height given by eq (76) is established, additional monomolecular layers of this step height will require the free energy given by eq (74), while any deviation from this value will require a free energy that includes the edge free energy. Then the distribution in step heights will be sharper than that calculated previously.

We have not considered explicitly the case where the monomolecular layer has a step height greater than the growing crystal face, but it can be shown that the rate of deposition of such a layer is negligible, if there is an appreciable increase in step height. Thus while the step height of the primary critical nucleus may persist for a time, it is expected that as the crystal grows the step height will be reduced to l* as given by eq (76) and the grown crystal will possess the step height l*.

The above remarks apply when the edge free energy, ***ϵ***, is not so large that the right hand side of eq (76) is larger than $l_{p}^{*}$, the step height of the critical nucleus. If, however, the value of l* as given by eq (76) is larger than $l_{p}^{*}$, then the grown crystal will have a step height $l_{p}^{*}$, characteristic of the homogeneously formed critical nucleus.

It should be mentioned that if the edge free energy in the primary nucleus, ***ϵ****_p_*, is included in our calculations it is found that

$$l_{p}^{*}=\frac{4\sigma_{e}}{\Delta f}+\frac{2C\epsilon_{p}}{\sigma_{s}}.$$ (77)

It must be understood that ***ϵ****_p_* and ***ϵ*** are in general different and in fact it is likely that ***ϵ*** is appreciably larger than ***ϵ****_p_*. Similarly, the free energy required to form a critical nucleus is

$$\Delta \phi_{p}^{*}=\frac{2C^{2}\sigma_{s}^{2}\sigma_{e}}{{\left(\Delta f\right)}^{2}}+\frac{2C^{2}\sigma_{s}\epsilon_{p}}{\left(\Delta f\right)}.$$ (78)

It may be stated in summary that, independent of the value of ***ϵ***, the grown crystal will have a step height that is quite uniform due to kinetic factors that arise from the nature of the saddle point in the free energy surface of forming stable growth nuclei. However, the step height of the grown crystal will be numerically different for different values of ***ϵ***: (I) If ***ϵ*** is negligibly small the step height of the grown crystal is given by eq (71). (II) If ***ϵ*** has a moderate value the step height of the grown crystal is given by eq (76). (III) If ***ϵ*** is very large the step height of the grown crystal is equal to that of the primary nucleus, and is given by eq (77). Case I can be distinguished from II and III by a determination of the melting point of these crystals that will be described in section 3.5. Cases II and III may be distinguished by an accurate measurement of the melting point of the crystals combined with an accurate measurement of the variation of step height with the temperature of crystallization.

In order that the overall kinetics of crystallization can be calculated, it is necessary to calculate **v***_c_*(*t, τ*), the volume at time *t* of a crystal that was nucleated at time *τ*. The volume growth in a crystal proceeds through the formation of a stable secondary nucleus on the growing (lateral) surface followed by the “crystallization” of the entire new molecule. The rate of addition of molecules to the crystal will equal the product of the average number of molecules in contact with the growing surface, ***ρ***, and the rate at which one of these molecules forms a stable nucleus, **p***_g_*. Then the rate of volume increase of the crystal will be.

$$\frac{dv_{c}}{dt}=aL\rho p_{g}$$ (79)

where **a*L*** is the average crystalline volume of a poly mer molecule. In section 2, the growth rate of the linear dimensions of a polymer crystal in a bulk phase was nucleation controlled and independent of time, unless impingements or chain entanglements between different crystals occurred. Impingements can be neglected in the growth of crystals in dilute solution. However, the growth rates are determined y both diffusion and nucleation processes, and are not in general independent of time for the loop nucleus. The number of polymer molecules per unit volume of the solution at the growing surface of the crystal, **n**(*t, τ*), will depend upon diffusion processes and the consumption of polymer molecules. Nevertheless, since only a rough estimate of the growth rate will be attempted, it will be assumed that

$$n(t,\;\tau )=n_{0}.$$ (80)

We can estimate ***ρ*** by assuming that every polymer molecule that approaches the edge of the crystal by a distance closer than one-half its mean end-to-end distance in solution, ***λ***, can form a secondary nucleus. Then, if ***λ***≫l*,

$$\rho =P(\pi \lambda^{2})\;n_{0}$$ (81)

where *P* is the perimeter of the crystal and **n**_0_ is taken as the average number of polymer molecules per unit volume at the growing surface of the crystal.

It is assumed that the shape of the cross section of the crystal is a parallelogram with an acute angle between two sides, *ψ*=70°. Experimental values of *ψ*=66° to 74° have been found by Till [3] for linear polyethylene crystals obtained from dilute solution. This is approximately the shape of the single crystals of polyethylene obtained by Keller [1]. It is also assumed that all four sides of the parallelogram have the same length, **X**, i.e., **X**=**x**=**y**. This assumption simplifies the following calculations, and is a consequence of the double spiral model used (it is clear for figs. 4b and 5a that **x**=**y**). Many other models also would lead to the same result. Then the volume of the growing crystal is

$$v_{c}=l*X^{2}\;\sin\;\psi .$$ (82)

The perimeter of the growing crystal is

P=4X. (83)

Combining eqs (79), (81), and (83),

$$\frac{dv_{c}}{dt}=4\pi \lambda^{2}aLp_{g}Xn_{0}.$$ (84)

Integration of eq (84) gives immediately

$$v_{c}(t,\;\tau )=aL+4\pi \lambda^{2}\mathrm{aL}p_{g}n_{0}\int_{\tau }^{t}Xd\tau^{\prime }.$$ (85)

Substitution of eq (82) into eq (84) yields after some manipulation

$$G=\frac{dX}{dt}=\frac{2\pi \lambda^{2}aL}{l*\sin\;\psi }p_{g}n_{0}.$$ (86)

Under the approximations employed above, the growth rate, **G**, of the sides of the crystal is independent of the time. Integration of eq (85) yields

$$X(t,\;\tau )=\frac{2\pi \lambda^{2}aL}{l*\sin\;\psi }p_{g}n_{0}(t-\tau )$$ (87)

where **X**(*τ,τ*) has been equated to zero.

Substituting eq (87) into eq (85) it is found that

$$v_{c}(t,\;\tau )=aL+\frac{8\pi^{2}\lambda^{4}{\left(aL\right)}^{2}{\left(p_{g}\right)}^{2}}{l*\sin\;\psi }\;{\left(n_{0}\right)}^{2}\;\frac{{\left(t-\tau \right)}^{2}}{2}.$$ (88)

Equation (74) gives us our desired result. It must be remembered that this equation is valid only in the early stages of the crystallization process, and only when diffusional effects are negligible. The term **a*L*** is the volume of the primary crystallite, and the second term represents the additional volume at time *t* due to accretion of new molecules on the lateral surfaces.

### 3.4. Value of **n** for the Overall Crystallization Process From Dilute Solution

In discussing crystallization from dilute solution we define the quantity, ***χ***, as the mass fraction of the total amount of polymer in the solution that is crystalline. ***χ*** will then be zero when no crystals have been formed, and will attain the value of unity if all the polymer present has entered the crystalline state. The crystalline mass is

$$M_{c}=\rho_{c}V_{c}$$ (89)

where ***ρ****_c_* and **V***_c_* are the density and volume of the crystalline phase. The total number of polymer molecules is **n**_0_**V***_s_*, where **V***_s_* is the initial volume of the solution and **n**_0_ is the initial number density of polymer molecules. If all polymer molecules were crystallized, the crystalline volume would be approximately **n**_0_**V***_s_***a*L***, where *L* is the mean length of a polymer molecule. Hence the total mass of polymer is

$$M_{\text{tot}}=\rho_{c}n_{0}V_{s}aL,$$ (90)

and by definition

$$\chi =\frac{M_{c}}{M_{\text{tot}}}=\frac{V_{c}}{n_{0}aLV_{s}}.$$ (91)

Since (**n**_0_**a*L*V***_s_*) is independent of time, the time dependence of ***χ*** is determined by the time dependence of **V***_c_*. The crystalline volume as a function of time is

$$V_{c}=V_{s}\int_{0}^{t}I\;(\tau )\;v_{c}\;(t,\;\tau )\;d\tau$$ (92)

where V*_s_****I***(*τ*)*dτ* is the number of stable nuclei formed between *τ* and *τ+dτ*, and **v***_c_*(*t, τ*) is the volume of a crystal that was nucleated at *τ*. From eqs (91) and (92).

$$\chi =\frac{1}{n_{0}aL}\int_{0}^{t}I\;(\tau )\;v_{c}\;(t,\;\tau )\;d\tau$$ (93)

Both the nucleation rate and the crystal growth rate will be reduced as crystallization proceeds due to the depletion of crystallizable material. Also **v***_c_*(*t*, *τ*) will be reduced in value if long range diffusion effects are important, and at the beginning of the crystallization process the nucleation rate ***I***(*τ*) will not have attained its equilibrium value. These circumstances introduce serious difficulties into an accurate evaluation of ***χ*** from eq (93). Instead of attacking these problems, we shall limit ourselves to the presentation of an approximate expression for ***χ*** in a form that has been widely used in the interpretation of experimental data.

In order to introduce this approximate expression, we define a new quantity, ***χ***′ which is the value of ***X*** that would result if all crystals were growing in a solution where the number density of uncrystallized polymer molecules remained at the constant value **n**_0_. From its definition it is clear that ***χ***′ may take on values from 0 to ∞. We shall assume that an adequate representation of the effect of the depletion of crystallizable material is given by

$$\frac{d\chi }{dt}=\frac{d\chi^{\prime }}{dt}\left[1-\frac{\chi }{X_{w}}\right].$$ (94)

Here **X***_w_* is the limiting value of ***χ*.** In dilute solution it is expected that **X***_w_* is very close to unity, and it will be assumed henceforth that **X***_w_*=1. (In the corresponding expression for bulk polymers [6], **X***_w_* can be considerably less than unity as a result of impingements). Equation (94) is clearly accurate at small values of t, and probably reasonably accurate up to moderate values of ***χ.*** From an integration of eq (94) it is found that

$$\chi =1-e^{-\chi^{\prime }}.$$ (95)

We have cast our expression for ***χ*** into this form for convenience in comparing our results with experimental data. Expressions of the form of eq (95) with ***χ***′ = **Z***_n_t^n^* have been derived phenomenologically by Mandelkern, Quinn, and Flory [9] and others [6], and have been widely used in interpreting experimental data. These expressions are plotted lor various integral values of *n* and comparison is made with experimental isotherms. The value of *n* which yields the best fit provides information concerning the geometry of the crystal growth. For example, if the crystals are nucleated sporadically in time and exhibit lineal two dimensional growth, *n* will be equal to three. It should also be noted that if ***χ***′ = **Z***_n_t^n^*, the isotherms defined by eq (95) obtained at various temperatures should be superposable simply with a shift in the time scale.

The quantity ***χ***′ is defined as that value of ***χ*** which would result if all crystals were growing in a solution of constant number density **n**_0_ of polymer molecules. Then the equilibrium nucleation rate is given by eq (51) and **v**_c_(*t*, *τ*) by eq (88). Substituting these values in eq (93) it is found that

$$\chi^{\prime }=Z_{1}t+Z_{3}t^{3}$$ (96a)

where

$$Z_{1}=\frac{kT}{h}e^{-\Delta F_{p}^{*}/kT}e^{-\Delta \phi_{p}^{*}/kT}$$ (96b)

$$Z_{3}=\frac{{\left(2\pi \lambda^{2}n_{0}\right)}^{2}}{3}-\frac{aL}{l*\sin\;\psi }\left(\frac{kT}{h}\right){\left(p_{g}\right)}^{2}e^{-\Delta F_{p}^{*}/kT}e^{-\Delta \phi_{p}^{*}/kT}$$ (96c)

where **p***_g_* is probably of the form given by eq (72).

It can be shown that the linear term (**Z**_1_*t*) often lies in an experimentally inaccessible region. Then

$$\begin{array}{l} \chi^{\prime }={\left(t\right)}^{3}\frac{\left(2\pi \lambda^{2}n_{0}\right)}{3}\frac{aL}{l*\sin\;\psi }\left(\frac{kT}{h}\right){\left(p_{g}\right)}^{2}e^{-\Delta F_{p}^{*}/kT}e^{-\Delta \phi_{p}^{*}/kT}. \end{array}$$ (97)

Substitution of eq (97) into eq (95) yields

$$\chi =1-e^{-Z_{3}t^{3}}$$ (98)

It is in order at this point to mention the principal approximations made in deriving eq (98) for simple loop-type crystals: (a) the depletion of crystallizable material was approximated by eq (94); (b) the growth of the crystals was assumed to be nucleation controlled instead of diffusion controlled; (c) the equilibrium nucleation rate was assumed to hold at all times; and (d) nucleation on the end surface of the crystals has been neglected.

The approximation for depletion of crystallizable material should be reasonably accurate for small and moderate values of ***χ***, although not valid for values of ***χ*** near unity. Since at sufficiently low concentrations of crystallizable material the crystal growth must become diffusion controlled, eq (98) cannot be accurate when ***χ*** is near unity. The validity of the assumption of nucleation controlled growth for low and moderate values of ***χ*** is more difficult to evaluate. It is believed reasonable by the authors that, except at very low concentrations of the crystallizable material, the effects of long range diffusion will not predominate. When these effects do predominate, the exponent of the time in eq (98) will be lowered somewhat. Finally, it is expected that the growth rate of the crystals is much more rapid than the primary nucleation rate. Under these circumstances the effects of the transient nucleation rate may be observed for low values of ***χ.*** This could cause the observed exponent of the time in eq (98) to be quite large for small values of ***χ***, even exceeding *n*=4. (In this region, the value of *n* is fictitious in the sense it does not reflect the type of nucleation and growth of the crystals.) If growth through secondary nucleation on the end surfaces is important the value of the exponent will be increased over what it would have been in the absence of such growth.

Our results may be summarized as follows: If the phenomenological expression

$$\chi =1-e^{-Z_{n}t^{n}}$$ (99)

is fitted to experimental data, we should expect that the best fit at moderate values of ***χ*** should be obtained for values of *n* near three. If long range diffusion limits crystal growth, somewhat lower values of *n* can be expected, whereas growth of the crystals through nucleation on the end surfaces will raise the value of *n.* At low degrees of crystallinity, higher values of *n* might be observed due to the effects of a transient nucleation rate. The value *n*=3 is, of course, that appropriate to (lineal) two-dimensional growth of objects born sporadically in time.

These results agree reasonably well with the experimental isotherms obtained dilatometrically by Mandelkern and Quinn [17, 18] on crystallization of polyethylene from a 0.25 percent solution of *α*-chloronaphthalene. Mandelkern has not investigated the morphology of the resulting polyethylene crystals, but he states that this concentration is comparable to that in which platelike crystals are formed. Superposable isotherms were obtained for crystallization temperatures from 97° to 104°C. The superposability of these isotherms is in marked contrast to the results he obtained with more concentrated solutions, but similar to that obtained for bulk crystallization. In addition, Mandelkern compared curves of the form of eq (99) with his isotherms and concluded that if the first 5 percent of the transformation is neglected, an almost exact fit is obtained for the major portion of the process if values of *n*=3 and *n*=4 are used. The first 5 percent of the crystallization process would require considerably higher values of *n* for a proper fit.

The agreement between our results and the experimental isotherms is consistent with the assumption that these isotherms result from the formation of crystals with folds. We shall proceed on this assumption and investigate the temperature dependence of the rate of overall crystallization in section 3.6. In order to perform this analysis we must estimate the equilibrium melting temperature, **T***_m_*. In estimating **T***_m_*, certain pitfalls can be avoided by elucidating some properties of crystals with folds that result from their metastability. This is done in the next section.

### 3.5. Metastability of Crystals Formed by Chain Folding

In previous sections the nucleation and growth of polymer crystals with loops has been discussed. We shall now give a brief treatment of the metastable character of these crystals. It will be demonstrated first that crystals with loops formed isothermally will have a relatively sharp melting point $T_{m}^{\prime }$, appreciably below the equilibrium melting temperature in the presence of solvent, **T***_m_.* The possibility that crystals with loops may have a tendency to increase their step height when stored at elevated temperatures will also be discussed. Before these points can be treated, it is necessary to consider the free energy difference between the crystalline state and the solution state for a crystal with loops.

If a crystal has ***v*** step elements of length **l**, its free energy with respect to the solution state is

$$\Delta \phi_{c}=2va\sigma_{e}+C\sigma_{s}\sqrt{\mathrm{va}}l-\mathrm{va}l\Delta f.$$ (100)

Equation (100) is formally identical with expressions for the free energies of nuclei that have been presented in previous sections, but several important distinctions must be noted. The crystal under consideration has been formed with a length **l** in an isothermal manner. We redefine the temperature of crystallization as **T***_x_*. Equation (100) represents the free energy of the crystal at a temperature *T*, which is not necessarily the same as the temperature of crystallization, **T***_x_.* The variation of the free energy of the crystal, **Δϕ***_c_*, with temperature is primarily due to the variation of the thermodynamic driving force, **Δf**, with temperature. The approximate variation of **Δf** with temperature was given in eq (28) which is rewritten here for convenience

$$\Delta f=\Delta h_{f}\left(\frac{T_{m}-T}{T_{m}}\right).$$ (101)

Finally, ***v*** is a very large number since eq (100) applies to a crystal, not a nucleus.

The volume of the crystal is ***v*al**. Then the free energy difference per unit volume of crystal between the crystalline and solution states is

$$\left(\frac{\Delta \phi_{c}}{v\mathrm{al}}\right)=\frac{2\sigma_{e}}{l}+\frac{C\sigma_{s}}{\sqrt{va}}-\Delta h_{f}\left(\frac{T_{m}-T}{T_{m}}\right).$$ (102)

Since the crystal has a large number of step elements, ***v****^−^***^½^** is small, and the second term on the right hand side of eq (102) will be neglected. Then

$$\left(\frac{\Delta \phi_{c}}{v\mathrm{al}}\right)=\frac{2\sigma_{e}}{l}-\Delta h_{f}\left(\frac{T_{m}-T}{T_{m}}\right).$$ (103)

The most stable crystal at any temperature will be that crystal which has a minimum value of ***Δϕ****_c_/****v*al**. It is clear from eq (103) that crystals with large step heights are more stable than crystals with smaller step heights. It is, of course, not surprising that a larger crystal is more stable than a smaller one. However, when a loop-type crystal of length **l** and of a given volume has been formed, it will probably be difficult for the step height to increase simply by having the crystal change its shape. Such an increase of step height would tend to be slow because of the complicated diffusion mechanism with lengthwise “sliding” of the polymer chains that would be involved. The ensuing discussion is carried out on the assumption that, in a melting experiment of sufficiently short duration, **l** will not increase appreciably.

If crystals with loops with length l* are formed isothermally at a temperature of crystallization, **T***_x_* they will melt at a temperature appreciably below the equilibrium melting temperature. In order to find the melting point we shall first derive an expression valid for any **l**. The temperature at which a crystal melts can be deduced from eq (103). A crystal with loops, which has a step height **l**, is stable at its temperature of formation with respect to the solution state. If, after the crystal was formed, the temperature is increased, the free energy increases. When the free energy of the crystal with respect to the solution state vanishes, the crystal will melt. Then the temperature of melting of a crystal with step height, **l**, is obtained by equating eq (103) to zero and solving for the temperature:

$$T_{m}^{\prime }(l)=T_{m}\left[1-\frac{2\sigma_{e}}{\Delta h_{f}l}\right].$$ (104)

We see that **T***_m_* is the melting temperature of a crystal with infinite step height.

The above expression, eq (104) has been derived with two tacit assumptions. It has been assumed that the rate of heating in the melting experiment is sufficiently rapid so that the step height, **l**, does not increase and sufficiently slow so that the actual melting temperature of the crystal will be observed to a close approximation. The recrystallization at greater step heights after melting need not be considered since the large negative temperature coefficient of the crystallization process ensures that recrystallization is very slow.

We have seen that in an isothermal crystallization the step heights of the crystals will be very close to a characteristic value, l*. Thus all the crystals formed in an isothermal crystallization will melt at almost the same temperature. This temperature, $T_{m}^{\prime }$, which is where these crystals melt, i.e., redissolve, is obtained by substituting the appropriate value of l* into eq (104):

$$T_{m}^{\prime }=T_{m}\left[1-\frac{2\sigma_{e}}{\Delta h_{f}l*}\right]$$ (105)

Let us first consider the case where the edge free energy, ***ϵ***, of nucleating a monomolecular layer is negligible. Then from eq (71) and eq (28)

$$l*=\frac{2\sigma_{e}}{\Delta h_{f}}\cdot \frac{T_{m}}{T_{m}-T_{x}}+\frac{kT_{x}}{C\sigma_{s}}.$$ (106)

Since *k***T***_x_*/*C****σ****_s_* is 20 A or less and l* is characteristically near 120 A, it follows that when eq (106) is substituted into eq (105), then $T_{m}^{\prime }$ is only a few degrees greater than **T***_x_*. Thus, if the edge energy is negligible, the crystals formed at a temperature of crystallization, **T***_x_*, will melt only a few degrees above **T***_x_.* Hence, an investigation of the temperature at which the crystals melt in solution can determine whether ***ϵ*** is negligible or not. The combination of these results with an accurate determination of the variation of step height 1* with the temperature of crystallization, **T***_x_*, should determine the importance of ***ϵ*** and ***ϵ****_p_.* If the step height of the crystal is as large as that of the critical primary nucleus with ***ϵ****_p_* neglected, then

$$l*=\frac{4\sigma_{e}}{\Delta h_{f}}\cdot \frac{T_{m}}{T_{m}-T_{x}}.$$ (107)

Substituting this value in eq (105) it is found that

$$T_{m}^{\prime }=\frac{1}{2}\left(T_{x}+T_{m}\right).$$ (108)

Then even if the step height is as large as that of a primary nucleus the crystal will tend to melt at a temperature approximately midway between the equilibrium melting temperature in the presence of solvent, and the temperature of crystallization.

The presence of a substantial number of crystallites with a small number of step elements would tend to broaden the melting curve, and imperfections due to branches might have a similar effect. It is to be understood that $T_{m}^{\prime }$ is to be measured under conditions where the warming rate is slow enough so that thermal equilibrium is established, but not so slow that l* has time to increase appreciably.

A direct determination of the equilibrium melting temperature in dilute solution, **T***_m_*, by slow warming may prove very difficult because of the persistence of the step height. The *“***T***_m_”* value so obtained could easily be somewhat low.

The above results were derived for crystals in solution, but it is believed that they would be qualitatively true if the crystals were removed from the solution and the solvent eliminated from the crystals. For example, a mass of dried loop-type crystals, previously initiated and grown to large **x** and **y** dimensions in an isothermal maimer from dilute solution at a temperature below **T***_m_*, should melt fairly sharply and well below **T***_m_*, the (bulk) equilibrium melting temperature.

It is not difficult to show that *a crystal with loops is more stable than a bundlelike crystal of the same size and shape* in a sufficiently dilute solution. The free energy difference between a loop-type crystal and the solution state is given in eq (100) The free energy difference between a bundlelike crystal of the same size and shape and the solution state is obtained by replacing **a, Δf**, ***σ***_s_, and ***σ***_e_ in eq (100) by a, Δ*f*_d_, *σ*_sd_, and (*σ*_ed_−*kT*log_e_*v*_2_/2*a*). The quantities a, Δ*f*_d_, and *σ*_sd_ are comparable to **a, Δf**, and ***σ***_s_. The end surface free energy for a bundlelike crystal in dilute solution was seen to be (*σ*_ed_−(*kT*log_e_*v*_2_/2*a*) in section 3.2, and in a sufficiently dilute solution this surface energy is greater than ***σ***_e_. Then in a sufficiently dilute solution the loop-type crystal is more stable than a bundlelike crystal of the same shape and volume, because the end surface energy contribution to the bundlelike crystal is much larger. In fact if the solution is sufficiently dilute so that loop-type nuclei are kinetically favored over bundlelike nuclei, the grown loop-type crystal are at the same time more stable than a bundlelike crystal of the same shape and volume. This result applies to crystals in solution.

If a loop-type crystal *of a given volume and cross-sectional shape* is in a dilute solution, the step height of this crystal will eventually approach an “equilibrium” value, where the total surface energy of the crystal will be minimized. The “equilibrium” value of the step height can be obtained by differentiating eq (100) with respect to **1** with the volume, ***v*al**, held constant, and then equating this result to zero. If the resulting equation is solved for **1**, it is found that the “equilibrium” value of the step height is (4***σ***_e_/(***Cσ***_s_)^2/3^***V***^1/3^, where ***V*** is the volume of the crystal. This result is based on the assumption that the polymer chains are much longer than the step heights considered. From this formula it follows that the “equilibrium” value of the step height is roughly equal to the lateral dimensions of the crystal. From this it is clear that the experimentally observed polyethylene crystals with characteristic step heights near 120 A have not attained their “equilibrium” step height through “sliding” diffusion.

### 3.6. Kinetics of the Overall Crystallization Process for Dilute Solution

In this section the theoretical expressions for the rate of overall crystallization are compared with the available experimental data with particular emphasis on the temperature dependence of the rate expressions. Unfortunately there are no available rate data in those systems where crystals with loops have been identified through morphological studies. The only accurate rate data for crystallization from dilute solution are the dilatometric measurements of Mandelkern and Quinn [17, 18] on the crystallization of linear polyethylene from a 0.25 percent solution of *α*-chloronaphthalene. The morphology of these crystals was not investigated, but Mandelkern states that the concentration range is comparable to that in which platelike crystals are developed. This encourages the belief that crystals with folds were predominant, especially since the temperature dependence of the shape of the isotherms is in marked contrast to results obtained for crystallization from more concentrated polymer solutions. An analysis will be performed on the assumption that crystallization through chain folding was predominant.

In preparation for an analysis of the experimental data a brief discussion will be given of the temperature dependence of the overall crystallization rates. Expressions for the overall crystallization rate were presented in eq (97) and (98) and will be rewritten here for convenience:

$$\chi =1-e^{-Z_{3}t^{3}}$$ (109)

where

$$Z_{3}=\frac{{\left(2\pi \lambda^{2}n_{0}\right)}^{2}}{3}\frac{aL}{l*\sin\psi }{\left(\frac{kT}{h}\right)}^{3}{\left(p_{g}\right)}^{2}e^{-\Delta F_{p}^{*}/kT}e^{-\Delta \phi_{p}^{*}/kT}.$$ (110)

When the temperature of crystallization is not too far from the melting point so that **ΔT** is small, the principal variation of **Z**_3_ with temperature is due to the factor $p_{g}^{2}\exp\left\{-\Delta \phi_{p}^{*}/kT\right\}$. The quantity $\Delta \phi_{p}^{*}$ has been previously obtained:

$$\Delta \phi_{p}^{*}=\frac{2C^{2}\sigma_{s}^{2}\sigma_{e}T_{m}^{2}}{{\left(\Delta h_{f}\right)}^{2}}\frac{1}{{\left(\Delta T\right)}^{2}}.$$ (111)

The temperature dependence of **p***_g_* is much smaller than $\exp\left\{-\Delta \phi_{p}^{*}/kT\right\}$. In fact it seems more likely that log **p***_g_* has a different temperature dependence than $\Delta \phi_{p}^{*}$. In any case we may write

$$Z_{3}=Z_{30}e^{-\frac{\alpha^{\prime }}{T{\left(\Delta T\right)}^{2}}},$$ (112)

where at low degrees of supercooling, **Z**_30_ varies slowly with temperature compared to exp {−*α*′/*T*(**ΔT**)^2^}. In the case where **p***_g_* does not contribute appreciably to *α*,′ we have

$$\alpha^{\prime }≅\alpha =\frac{2C^{2}\sigma_{s}^{2}\sigma_{e}T_{m}^{2}}{{\left(\Delta h_{f}\right)}^{2}k}.$$ (113)

For the remainder of this section it will be assumed that eq (113) is valid, although this is not essential to our analysis.

It has been shown that eq (109) is an adequate description of the isotherms of crystallization at moderate values of ***χ*** for the crystallization of linear polyethylene from a dilute solution of *α*-chloronaphthalene. If the temperature of crystallization is changed, the value of **Z**_3_ is changed. The shape of the isotherm remains unchanged although the time scale is shifted. This allows us to specify the rate of the crystallization process by the time required for the value of ***χ*** to reach 0.5. Then from eq (109)

$$Z_{3}{\left(t_{1/2}\right)}^{3}=\log_{e}2.$$ (114)

If the logarithm to the base 10 is taken of both sides, and eq (112) is substituted into the result, it is found after some manipulation that:

$$\log_{10}\left[\frac{1}{t_{1/2}}\right]=\frac{1}{3}\log_{10}\left[\frac{Z_{30}}{\log_{e}2}\right]-\frac{1}{3}\frac{\alpha \log_{10}e}{T{\left(\Delta T\right)}^{2}}.$$ (115)

It is clear from eq (115) that if experimental values of log_10_(l/*t*½) were plotted against *T*^−1^**(ΔT**)^−2^ for various crystallization temperatures, an approximately straight line should be obtained. The value of the product (***σ****_s_^2^****σ****_e_*) could be obtained from the slope of this straight line. Since **ΔT=T***_m_*−T, it is clear that the equilibrium melting temperature, **T***_m_*, must be known before such a plot could be constructed.

**Figure 9:**
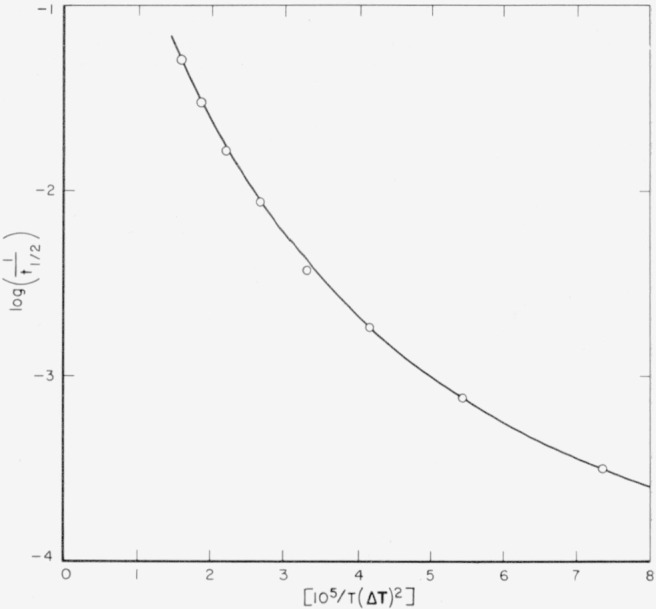
Plot of log (1/t_½_) against [10^5^/T(**ΔT**)**^2^**] for a 0.25 percent polyethylene solution in α-chlorcnaphthalene with **T**_m_=110° C. (After Mandelkern, see ref. [18]).

Mandelkern and Quinn [17, 18] have observed the isothermal crystallization of linear polyethylene from a 0.25 percent solution of *α*-chloronaphthalene dilatometrically. The shapes of their isotherms agree at values of moderate ***χ*** with eq (109), so that it seems reasonable to apply eq (115) to the temperature dependence of these isotherms. Mandelkern has tabulated the values of *t*_½_ for one degree intervals of the crystallization temperature between 97° C and 104° C. He also quotes the equilibrium melting temperature as being between 109° C and 110° C [18], and presents a plot of log(1/*t*_½_) versus 100/(**ΔT**)^2^ which is based on this value of **T***_m_.* In figure 9 a similar plot is presented. Figure 9 is constructed from the tabulated values of Mandelkern, plotting log(1/*t*_½_) against 10^5^/*T*(**ΔT**)^2^ with **T***_m_*=110° C. The curve shown in figure 9 is certainly not a straight line, but is rather concave upwards. Moreover, the slope at the lower degrees of supercooling is smaller in magnitude than the corresponding slope for bulk polyethylene which would appear to indicate that ***σ****_s_^2^****σ****_e_* is smaller than the corresponding product for the bulk polymer. These facts stand in apparent contradiction to the theory presented in this paper.

The morphology of the crystals was not investigated by Mandelkern and Quinn. It is therefore possible that the theory developed in this paper is not applicable to the data plotted in figure 9. However, it is no easier to explain the curvature in figure 9 if one assumes that *bundlelike* crystals were nucleated either homogeneously or heterogeneously. Since the deviation from a straight line of log(1/*t*_½_) plotted against *T*^−1^**ΔT**^−2^ or *T*^−1^**ΔT**^−1^ is not accounted for by the hypotheses just given, a further discussion of the data will be given. This discussion will show that the experimental data are not necessarily inconsistent with the theory presented in this paper and will serve the useful purpose of emphasizing the care required in obtaining experimental evidence that provides a critical test of this theory.

**Figure 10:**
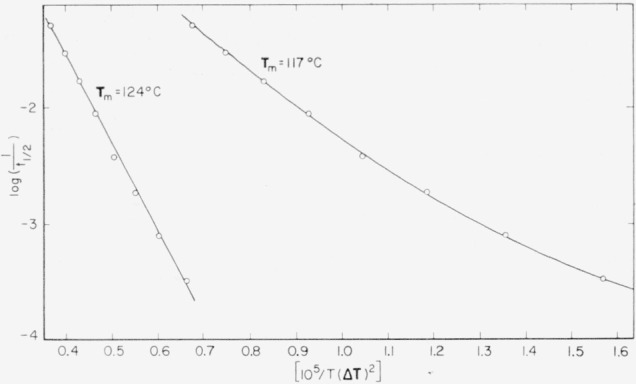
Plot of log (1/t_½_) against (10^5^/T(**ΔT**)**^2^**) for 0.25 percent polyethylene solution in α-chloronaphthalene for two assumed values of **T**_m_ showing approach to straight-line behavior. The slope of the line obtained with **T**_m_=124° C implies a $\sigma_{s}^{2}\sigma_{e}$ value of ~1070 erg^3^ cm^−6^.

Upon reflection, a possible resolution of the apparent discrepancy can be seen. Suppose that the value **T***_m_*=110° C was obtained by dilatometrically observing the melting of the crystals in the solution. In the previous section it was shown that crystals formed in dilute solution may melt sharply well below the equilibrium melting temperature for dilute solution. Then the correct value of **T***_m_* might be appreciably higher than 110°. Rough estimates of **T***_m_* can be made by two separate methods. First, if it is assumed that eq (108) is accurate, and that the observed melting temperature, $T_{m}^{\prime }$, is 110° C, the equilibrium melting temperature is obtained if the crystallization temperature is given. For example, if it is assumed that for a sample crystallized at 96° C the observed melting temperature of these crystals is 110° C, then **T***_m_*=124° C. If the temperatures of crystallization and observed melting were 103° and 110° C, **T***_m_*=117° C. This method of estimating **T***_m_* has two drawbacks: (a) the estimate furnishes a range of values of **T***_m_* instead of a single value, and, (b) eq (108) is probably not very accurate. Another method of estimating **T***_m_* is to plot log_10_(1/*t*_½_) against (10^5^/*T*(**ΔT**)^2^) for various values of **T***_m_.* **T***_m_* is taken to be that value which yields a straight line plot, if such a value exists. This method of estimating **T***_m_* is based on the correctness of eq (115). In figure 10 plots of log (1/*t*_½_) against (10^5^/*T*(**ΔT**)^2^ are presented for the assumed values of **T***_m_*=117° C and **T***_m_*= 124° C. The plot for **T***_m_*=117° C has considerable curvature, but the plot for **T***_m_*=124° C is fitted well by a straight line.

It is found from the slope of the plot in figure 10 for **T***_m_*=124° C that $(\sigma_{s}^{2}\sigma_{e})=1070$. This value is much larger than the corresponding value obtained from the data on bulk polyethylene where $\left(\sigma_{s}^{2}\sigma_{e}\right)$ ~ 100, as calculated from the slope of the plot of (*log* 1/*t*_½_) against 100/(Δ*T*)^2^ presented by Mandelkern [18]. This is consistent with the concept that ***σ****_e_*>*σ_e_*, eq (27). Then the supposition that **T***_m_*= 124° C resolves each of the apparent discrepancies between the theory presented in this paper and the experimental rates. It is, of course, not clear that the plot of log (1/*t*_½_) should be exactly straight since **Z**_30_ in eq (115) is temperature dependent. However, even if **T***_m_* is as low as 117° C the plot in figure 10 corresponds to a value of $\sigma_{s}^{2}\sigma_{e}$ which is larger than that observed for the bulk polymer. It is clear that there is no inconsistency between this data and the theory presented in this paper if **T***_m_* is appreciably larger than 110° C.

Such high values of **T***_m_* are not inconsistent with the errors in estimating **T***_m_*. In determining the heat of fusion of polyethylene, Quinn and Mandelkern [14] measured the melting temperature of polyethylene as a function of concentration in various solvents. The heat of fusion per mole of repeat units, Δ*H_u_*, was calculated by fitting the experimental data to the equation

$$\frac{1}{T_{m(d)}}-\frac{1}{T_{m}}=\frac{R}{\Delta H_{u}}\left[\frac{V_{u}}{V_{1}}\right]\left\{v_{1}-\frac{BV_{1}}{R\;T_{m(d)}}v_{1}2\right\}.$$ (116)

Here *T_m_* is the equilibrium melting temperature of the bulk polymer, *V_u_* and *V*_1_ are the molar volumes of the repeating unit and the diluent respectively, *B* is the interaction energy density characteristic of the solvent solution pair, and *T_m_*_(_*_d_*_)_ the equilibrium melting temperature in the presence of diluent. (We adhere to the assumption that a value of *T_m_*_(_*_d_*_)_ for *v*_1_→1 obtained under equilibrium conditions is very close to **T***_m_*.) While Δ*H_u_* was determined within a few percent, Quinn and Mandelkern state that *B* could be in error by several cal·cm^−3^. For polyethylene in *α*-chloronaphthalene they obtain a value *B*≅0. If a value of *B*= 2 cal·cm^−3^ were assigned and Δ*H_u_* left unchanged *T_m_*_(_*_d_*_)_→124° C as *v*_1_→1. Thus *T_m_*_(_*_d_*_)_=124° C lies within the assigned experimental error. On the other hand it is somewhat difficult to reconcile the data of Quinn and Mandelkern for moderate concentrations of polyethylene in *α*-chloronaphthalene with a value of *T_m_*_(_*_d_*_)_ for *v*_1_→1 as high as 124° C. In short, no definite conclusion can be drawn, but the authors feel that the apparent discrepancy may arise from an incorrect value of **T***_m_.*

Even if the correct value of **T***_m_* is 110° C for a dilute solution of polyethylene in *α*-chloronaphthalene, the data of Mandelkern and Quinn are not necessarily inconsistent with the theory presented in this paper. Equation (115) was derived on the assumption that the equilibrium nucleation rate was attained. If, however, the growth rate of the crystals is so great compared to the nucleation rate that the early nucleation transient determines the overall crystallization rate, a different type of expression may be expected. No attempt will be made to obtain an accurate expression for the transient nucleation rate, but the influence of $\Delta F_{p}^{*}$, the free energy barrier to addition of another step element, will be very pronounced. This would lead to a plot of log (1/*t*_½_) versus *T*^−1^**ΔT**^−2^ similar to that shown in figure 9. It should be noted that the first 5 percent of the isotherms obtained by Mandelkern seemed to imply a transient nucleation rate. It should also be noted that if transient nucleation is determining the rate of crystallization of crystals with loops, a reduction in the concentration of the solution will reduce the growth rate and straighten the plot of log (1/*t*_½_) versus *T*^−1^(**ΔT**) ^−2^. The curvature of the plot obtained by Mandelkern and Quinn could apparently be explained if ***ϵ****_p_* were not negligible because then $\Delta \phi_{p}^{*}$ would be given by eq (78) and hence a straight line plot would not be expected. This, however, does not account for the low value of $\sigma_{s}^{2}\sigma_{e}$ obtained from their data when **T***_m_* is 110° C. The data of Mandelkern and Quinn may also be explained by other special assumptions, but these possibilities do not appear to be as likely as the ones cited.

It is clear that for a proper evaluation of the experimental data an accurate value of **T***_m_* must be obtained. Since it has been shown in section 3.4 that crystals with loops may melt well below equilibrium melting temperature, it is possible that this represents a serious problem for dilute solutions. One possibility is to measure the temperature of melting and the characteristic step height, l*, as a function of crystallization temperature and attempt to extrapolate to the equilibrium melting temperature by the use of eq (105).

## 4. Discussion

### 4.1. Brief Summary of Results

The general predictions yielded by the present study can be summarized in the following manner:

When a crystallizable linear polymer is precipitated from sufficiently dilute solution by supercooling, platelike crystals with a definite step height l* will form. In these crystals, the chain axes of the polymer molecules will be perpendicular to the two large flat faces of the platelike crystals. The aforementioned flat faces will contain chain folds, i.e., they will consist of loops. The step height depends on the temperature of crystallization, and on the surface free energy, ***σ****_e_*, of the interface containing the loops. The step height is larger for higher crystallization temperatures, and increases with ***σ****_e_.* The latter quantity will be fairly large, owing to the fact that the work required to form a loop is involved. (The corresponding quantity for bundlelike nuclei, ***σ****_e_*, is considerably smaller since it contains no loop energy.) At the degree of supercooling commonly encountered in practice, **ΔT** = 10 to 40° C, 1* may be expected to lie in the range 50 to 500A. The most perfect crystals will be formed from highly dilute solution, and with unbranched polymer. More imperfect specimens will be formed from more concentrated solution, and a threshold will be reached where very poor crystals will form. The step height will be remarkably uniform if the crystallization is carried out isothermally from a highly dilute solution. Pyramidal growth, where one crystal with fixed step height grows on the flat (loop containing) face of another, is to be expected at moderate dilution, but single crystals should be common at low concentration. In exceptional cases, crystals consisting of but one molecule may be observed. Much more common will be crystals that have grown to fairly large dimensions by successive addition of new polymer molecules through secondary nucleation on the lateral surfaces. These will have substantially the same step height as the primary crystallite. In many cases, distinct protrusions on the lateral surfaces due to secondary nucleation and growth may be seen. The more perfect crystals will often have a regular shape of simple geometric form when viewed normal to the surface containing the loops. Depending on the crystal system, the crystals could, for example, be diamond- or hexagonshaped.

The overall rate of crystallization will probably follow a law where *n*=3 or *n*=4, most likely nearer the former, over the main part of the process, but deviations from the suggested range in the early and late stages are a distinct possibility. In the early stages a steady-state rate of nucleation may take some time to develop, and in the late stage, where the majority of the molecules have already been swept from the solvent, *n* may fall.

The crystals containing chain folds formed in dilute solution are metastable: Even in the case where a crystallite of step height l*, which is formed in solution at a crystallization temperature, **T***_x_*, is allowed to grow to very large size in the other two dimensions, it will still melt appreciably lower than **T***_m_*, the melting temperature in the presence of solvent of a crystallite that is large in all three dimensions. (Crystals free of solvent formed by drying crystals with loops formed in dilute solution will behave in a qualitatively similar manner, and melt well below the equilibrium bulk melting temperature, *T_m_.* The observed melting point, $T_{m}^{\prime }$, of a set of crystailites formed isothermally from dilute solution may be surprisingly sharp (but low) owing to the uniformity of the step height. This will be especially true for large crystals precipitated from very dilute solution. If a set of crystals with loops with characteristic step height $l_{1}^{*}$, is formed at an isothermal crystallization temperature *T*_1_, and then the temperature of the solution raised to *T*_2_, where the characteristic step height is $l_{2}^{*}$, $l_{1}^{*}$ will still tend to persist for some period of time at *T*_2_. Thus the melting point $T_{m}^{\prime }$ characteristic of $\left(l_{1}^{*},\;T_{1}\right)$ will tend to persist even though the temperature of the solution is raised. The equilibrium melting temperature of crystals with loops in dilute solution may thus be very difficult to determine accurately in some cases by the conventional method of slowly raising the temperature.

An isothermal increase of **l** due to “sliding” type diffusion in the crystal may occur.

The temperature dependence of the rate of nucleation for nuclei with loops should follow a law of the general form In (***I***/***I***_0_)∝***α***/*T*(**ΔT**)^2^. The value of $\sigma_{s}^{2}\sigma_{e}$ that may be estimated from ***α*** should be larger than the value of $\sigma_{s}^{2}\sigma_{e}$ obtained for the bundlelike nuclei characteristic of homogeneous bulk nucleation in the same polymer. In order to test the temperature dependence of ***I***/***I***_0_, it is necessary to have a reliable value of **T***_m_*, so that **ΔT** is known accurately.

### 4.2. Crystals With Loops in Bulk Polymers, and Heterogeneities

The theory given in the foregoing sections deals with *homogeneous* initiation of loop-type crystals in dilute solution. The theory renders it clear that near and below some threshold value of the concentration, that loop-type nuclei will begin to predominate, provided that loop formation is sterically feasible. The theory does not attempt to predict what type of crystal might tend to form in an intermediate concentration range where bundlelike and loop-type nuclei compete. We have indicated that in crystallizable linear polymers in bulk that the conventional bundlelike nucleus seems highly probable. It should be clearly understood that what is meant here is that bundlelike nuclei of *homogeneous* origin seem probable in such bulk polymers; this is not necessarily related to what type of nucleus might form by *heterogeneous* nucleation on the surface of a wettable foreign particle. Moreover, we do not incline to the view that crystals with loops are impossible to form by a homogeneous process in a bulk polymer, and this subject, though obviously speculative, deserves brief discussion.

In a bulk polymer, where *v_2_*= 1, bundlelike homogeneous *nuclei* should certainly predominate if ***σ****_e_*>***σ****_e_.* (There is little reason to expect that the free energies of activation controlling the jump-rate at the super-cooled-liquid—crystal interface would be such as to cause a preponderance of loop-type nuclei in bulk.) Then if bundlelike nuclei can grow, the polymer will crystallize without loop formation. However, if the radial growth of the bundlelike nucleus is severely impeded by the strain effect mentioned in section 2.2, which results from the increasing difference in the lattice spacing of the crystal and “liquid” just outside the ends of the bundlelike nucleus as it grows radially, actual crystallization resulting from such nuclei may be greatly subdued. Then another crystallization process may enter. Since according to our formulation, the formation of a few loop-type nuclei is possible at *v*_2_=1, and in view of the fact that these would grow if crystallizable material were present, the majority of crystallites actually observed in the bulk phase in such a case would be of the loop-containing variety. The hypothesis that bundlelike nuclei may be prevented from growing to large size by strain, coupled with the reasonable belief that loop-type nuclei, once formed, might not be subject to such a strain effect on growth, thus leads to the possibility that loop-type crystals could make up the main part of the crystallization in the bulk phase. Even then, numerous bundlelike nuclei would be present. The main point of the present theory, however, is that loop-type nuclei (and subsequently crystals derived from them) are quite certain to appear at sufficiently great dilution, provided that loop formation consistent with crystal structure is sterically possible. The theoretical prediction of homogeneously induced loop-type crystals in bulk depends on additional factors, and is altogether more of an open question.

At various places in the literature, evidence has been given suggesting that crystals with folds may arise in bulk polymers (see ref. [1]). The presently available evidence that such “structures” as are seen in bulk polymers may be associated with a step height resulting from nuclei with chain folds that are of *homogeneous* origin is incomplete. If it is in fact true that step structures associated with folds actually exist in the bulk phase, we believe full consideration must be given to the possibility that heterogeneities or surfaces may be involved. We consider it possible that nuclei with folds may form at the surface of a heterogeneity in a bulk phase, some or nearly all of the energy deficit arising from the bending energy **q** being made up by the interaction energy of the polymer molecule with the heterogeneity. Also, special structures may tend to develop at the surface of a bulk polymer specimen.

From a theoretical point of view, very considerable confusion can be caused by assuming that any structure seen in a bulk polymer, or on its surface, is a result of homogeneous initiation. It is now known that quite stringent measures are frequently required to develop the intrinsic nucleation mechanism in a bulk polymer. For example, careful filtration and selection of samples coupled with strong superheating prior to crystallization is evidently advisable in some instances. Precautions of the type just mentioned, which are designed to enhance the homogeneous nucleation mechanism, do not seem to be commonly employed in morphological studies on bulk polymers.

Our views concerning the existence of loop-type crystals in bulk polymers may be summarized as follows: (a) While the evidence that loop-type crystals exist in bulk polymers is mounting, proof that such crystals are of homogeneous origin is lacking; (b) if such loop-type crystals are in some polymer proved to be of homogeneous origin, consideration should be given to the possibility that strain subdues or prevents the growth of bundlelike nuclei and, since a few loop-type nuclei will be present, thus allow the predominant crystalline form to possess loops; (c) a likely source of loop-type nuclei is a heterogeneity, and full consideration must be given to this fact in interpreting data on bulk polymers that have not been subjected to special treatment; (d) a proof that crystals with chain folds occurred in a bulk polymer by either homogeneous or heterogeneous initiation would not invalidate the general approach here for the formation of loop-type nuclei and crystals from dilute solution.

With regard to the effect of heterogeneities in dilute solution, it is clear that they will accelerate the crystallization process. However, by careful filtration, centrifugation, or previous precipitation, it should be possible to eliminate the effect of foreign bodies to a degree sufficient to permit the intrinsic mechanism to manifest itself. Judging from the remarks of Keller and O’Connor [1] concerning their technique and results, it would appear that many of the crystals that they discussed were formed in the body of the solution, and not on motes in the solution, or on the container walls. There is also reason to believe that some of the other work cited, notably the rate studies of Mandelkern [17, 18], may refer to homogeneous initiation. Nevertheless, it is mandatory to exercise considerable care in carrying out rate experiments in dilute solution in such a way as to subdue the effect of foreign bodies.

### 4.3. Concluding Remarks

In this paper, a point of view is expressed that leads to a number of definite predictions concerning the formation of polymer crystals with loops from dilute solution. Perhaps the most important and compelling prediction is that crystals of this type will be deposited from sufficiently dilute solution if it is physically possible to form a fold that is consistent with lattice structure and steric considerations. Definite predictions are also given, under certain assumptions, concerning the variation of step height with crystallization temperature, metastability and melting point, the *n* law connected with the appearance of loop-type crystals from dilute solution, the temperature dependence of the kinetics of crystallization, and the constancy of step height in a crystal grown in an isothermal manner. In addition, rough numerical estimates of important fundamental quantities, such as ***σ****_e_*, are given. Insofar as it can be tested, the theory seems to be in at least approximate accord with the facts presently known. It is believed that the theory is sufficiently to the point as to provide a reasonable framework for future experimental studies even if it proves not to be quantitatively correct. Moreover, specific experimental approaches, together with their attendant (and sometimes formidable) difficulties are mentioned. No claim is made that the theory is complete. For example, it is obvious that the interesting details of the structure of the fold itself have been largely passed over, and some of the possibilities concerning the growth mechanism which could, for instance, lead to a ramp-type of growth due to spiral dislocations have not been mentioned.

## Figures and Tables

**Figure 11:**
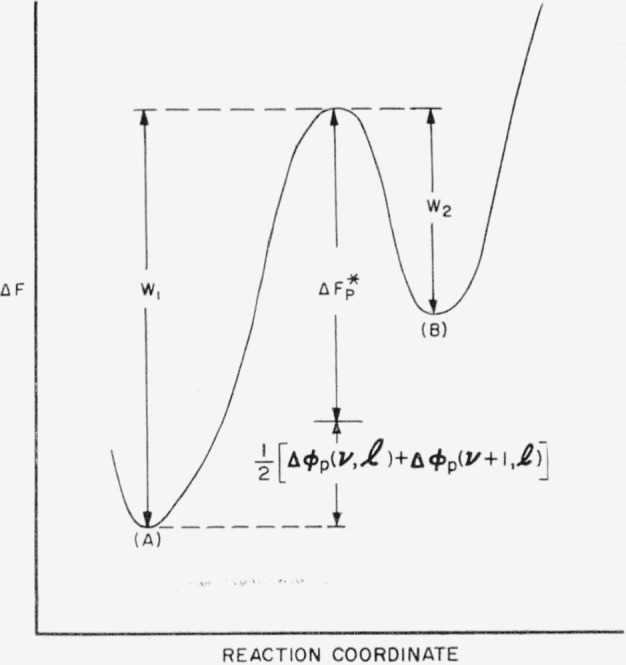
Cross section of free energy surface for the most probable reaction path between embryos of **v** step elements o length **l** and embryos of (**v** + 1) step elements of length **l.** The energy minima at (A) and (B) represent the free energy difference from the liquid state of the two different embryos, respectively.

**Table 1:** Values of n and Z_n_ for various modes of growth of bundlelike nuclei (homogeneous nucleation)

Mode of growth	*n*	Form of bulk rate constant^a^
		
One-dimensional	2	*Z*_2_∝*IG_l_*
Two-dimensional	3	*Z*_3_∝*IG_r_*^2^
Three-dimensional^b^	4	*Z*_4_∝*IG_l_IG_r_*^2^

a In these expressions *I* may refer to either *I_A_* or *I_B_.*

b If branches develop, and *Z*_4_∝*IG*^3^*_sph_*., where *G_sph_*. is the mean growth rate in the radial dimension.

## Footnotes

1 Figures in brackets indicate the literature references at the end of this paper.

2 Even if it is assumed that the nucleus is an ellipsoid of revolution, an expression for *I_A_* similar to eq (7) is obtained. (See S. Matsuoka and B. Maxwell, Plastics Laboratory Technical Report 53E, Princeton University, 1959.) When the nucleus is large, i.e., at Low supercooling, the ratio of the major and minor axes is determined by *σ_s_/σ_e_*. However, such a nucleus will tend to take on the shape of a disk or parallelepiped as the degree of supercooling is increased so that region *B* is approached. Thus, the overall behavior of *I_A_*, including its temperature dependence and transition to region *B*, is unaffected by assumptions concerning the shape of the nucleus in region *A.*

3 The relation Δ*f*=Δ*hf*Δ*T/T_m_* is usually employed to give the free energy difference between the supercooled liquid and crystalline states. This expression is not as precise for a glass forming system as eq (10).

4 Over long periods of time, the step height will probably gradually increase in an isothermal process by lengthwise diffusion of segments. This point is treated more fully later.

5 The authors are indebted to Dr. C. W. Beckett for a helpful discussion concerning the probable value of **q**.

6 It is quite possible that local rearrangements of the segments would cause the step elements in the nucleus to become even more uniform in a relatively short period of time.

7 The Turnbull-Fisher theory is not applicable when the activated state is reached in one step, as in the present case of a monomolecular layer. The treatment of the nucleation rate in appendix 5.2 deals with the situation where the growth nucleus is formed in one step.

## 5. Appendix

### 5.1. Equilibrium Nucleation Rates of Crystals With Chain Folds

We wish to calculate the equilibrium nucleation rate per unit volume of crystals formed by chain folding. The method of derivation, which is merely outlined below, is that used by Turnbull and Fisher [8]. The reader is referred to that paper for details.

A nucleus of ***v*** step elements of length, **l**, can gain or lose a step element by an elementary process. This nucleus may be specified by **l** and ***v*** where ***v*** may possess the values

$$v_{m},v_{m}+1,v_{m}+2,\ldots .$$

***v****_m_* is the minimum number of step elements in a nucleus. The number of nuclei per unit volume with ***v*** segments and a length between **l** and **l**+**d**l is *η*(***v***,**l**)*d***l**. The free energy of a primary embryo or nucleus is

$$\Delta \phi_{p}(v)=(C\sigma_{s}l\sqrt{a})\sqrt{v}-[l\Delta f-2\sigma_{e}]av.$$ (A-1)

This function has a maximum value at

$$v^{‡}=\frac{{\left(C\sigma_{s}l\right)}^{2}}{4a{\left(l\Delta f-2\sigma_{e}\right)}^{2}}.$$ (A-2)

The value of Δ*ϕ_p_* when expanded about ***v***^‡^ is

$$\Delta \phi p=\Delta \phi_{p}^{‡}-\frac{a^{2}{\left(l\Delta f-2\sigma_{e}\right)}^{3}{\left(v-v^{‡}\right)}^{2}}{{\left(C\sigma_{s}l\right)}^{2}}+\ldots$$ (A-3)

where

$$\Delta \phi_{p}^{‡}=\frac{{\left(C\sigma_{s}l\right)}^{2}}{4(l\Delta f-2\sigma_{e})}.$$ (A-4)

A nucleus of length **l** can only become stable by addition of step elements. **l** cannot vary without dissolution of the minimum size nucleus. The free energy diagram for nuclei of ***v*** and ***v+***1 step elements with some fixed length **l** is shown in figure 11. The rate that nuclei with ***v*** step elements become nuclei with ***v+***1 step elements is

$$\eta (v,l)dl\left(\frac{kT}{h}\right)\exp\left\{-\frac{W_{1}}{kT}\right\},$$

whereas the rate of the backward reaction is

$$\eta (v+1,l)dl\left(\frac{kT}{h}\right)\exp\left\{-\frac{W_{2}}{kT}\right\}.$$

Then the net rate of nucleation *i*(**l**)*d***l** is

$$i(l)dl=\left(\frac{kT}{h}\right)\left[\eta (v,l)dl\;\exp\left\{-\frac{W_{1}}{kT}\right\}-\eta (v+1,l)dl\exp\left\{-\frac{W_{2}}{kT}\right\}\right].$$ (A-5)

Now

$$\begin{array}{l} W_{1}=\Delta F_{p}^{*}+\frac{1}{2}\left[\Delta \phi_{p}(v+1,l)-\Delta \phi_{p}(v,l)\right]\\ W_{2}=\Delta F_{p}^{*}+\frac{1}{2}\left[\Delta \phi_{p}(v+1,l)-\Delta \phi_{p}(v,l)\right], \end{array}$$ (A-6)

where $\Delta F_{p}^{*}$ is the free energy barrier associated with addition or subtraction of a segment from the nuclei. The value of $\Delta F_{p}^{*}$ will be independent of ***v*** and probably independent of **l**.

Now if ***v*** is a large number we are justified in treating ***v*** as a continuous variable. With this approximation eqs (A-5) and (A-6) become

$$i(l)dl=-\frac{kTdl}{h}\left\{\frac{\partial \eta }{\partial v}+\frac{\eta }{kT}\frac{\partial (\Delta \phi_{p})}{\partial v}\right\}e^{-\Delta F_{p}^{*}/kT}$$ (A-7)

where higher derivatives with respect to ***v*** have been neglected. This equation is easily integrated from ***v****=s* to ***v***=∞. Using the boundary condition *η*(∞, **l**) = 0 for the equilibrium case we have

$$i(l)=\frac{kT}{h}\frac{\left[\eta_{s}e^{\Delta \phi_{p}(s)/kT}\right]}{\int_{s}^{\infty }d\;ve^{\Delta \phi_{p}(v)/kT}}e-\Delta F_{p}/kT.$$ (A-8)

The right hand side of eq (A-8) may be evaluated if s is chosen so ***v****_κ_<<s*<<***v***^‡^. We find then by use of eq (A-3) that

$$\int_{s}^{\infty }dv\;e^{\Delta \phi_{p}(v)/kT}≅{\left[\frac{a^{2}{\left(l\Delta f-2\sigma_{e}\right)}^{3}}{\pi {\left(C\sigma_{s}l\right)}^{2}kT}\right]}^{-1/2}e^{\Delta \phi_{p}^{‡}/kT}$$ (A-9)

which is a good approximation when $\Delta \phi_{p}^{‡}/kT>>1$.

We may evaluate *η*(*s*,**l**) if *s* is sufficiently small that a Maxwellian distribution holds. Then

$$\eta (s,l)dl=Adl\;e^{-\Delta \phi_{p}(s)/kT}.$$ (A-10)

If *s* is sufficiently small we may assume that only the surface energy terms are important. Then

$$\Delta \phi_{p}≅\Delta \phi_{p}^{\prime }=(C\sigma_{s}l\sqrt{a})\sqrt{v}+2\sigma_{e}av.$$ (A-11)

Since the sum over all states *s*,**l** must equal the number of polymer molecules per unit volume we may evaluate *A.* Treating *s* and **l** as continuous variables running from zero to infinity

$$A=n_{0}{\left\{\int_{0}^{\infty }dv\int_{0}^{\infty }dl\;e^{-\Delta \phi_{p}^{\prime }/kT}\right\}}^{-1}.$$ (A-12)

Substitution of eq (A-11) into (A-12) gives us

$$\eta (s,l)\;e^{\Delta \phi_{p}(s)/kT}=\left[\frac{C\sigma_{s}a{\sqrt{2\sigma }}_{e}}{\sqrt{\pi }{\left(kT\right)}^{3/2}}\right]n_{0}.$$ (A-13)

Then substituting (A-13) and (A-9) into (A-8) we have the desired expression

$$i(l)=\left[\frac{a^{2}{\sqrt{2\sigma }}_{e}{\left(l\Delta f-2\sigma_{e}\right)}^{3/2}}{\pi {\left(kT\right)}^{2}l}\right]\frac{n_{0}kT}{h}e^{-\Delta F_{p}^{*}/kT}\;e^{-\Delta \phi_{p}^{‡}/kT}.$$ (A-14)

We may calculate the total nucleation rate by integrating eq (A-14) over all **l**. The number of stable nuclei formed per unit volume of solution per unit time is

$$I=K\frac{n_{0}kT}{h}e^{-\Delta F_{p}^{*}/kT}\;e^{-\Delta \phi_{p}^{*}/kT}$$ (A-15)

where

$$\Delta \phi_{p}^{*}=\frac{2C^{2}\sigma_{s}^{2}\sigma_{e}}{{\left(\Delta f\right)}^{2}}$$ (A-16)

$$K=\frac{{\left(2\sigma_{e}\right)}^{3/2}\;(\Delta f)a^{2}}{\sqrt{\pi }C\sigma_{s}{\left(kT\right)}^{3/2}}$$ (A-17)

Throughout this appendix it has been assumed that each nucleus is composed of step elements of uniform length. This assumption has simplified the derivation of eq (A-15) and has led to an explicit expression for the distribution of the lengths of the step elements in stable nuclei in eq (A-14). It is, of course, possible that an embryo or nucleus could be composed of step elements of different lengths. The remainder of this appendix is devoted to discussing this more general case. This discussion will support the validity of the above results.

In the general case an embryo or nucleus will have ***v*** step elements which have lengths: **l**_1_ **l**_2_, … **l***_v_*. The principal difficulty in treating this case is in obtaining and handling appropriate expressions for the free energy of such nuclei. Fortunately for the purposes of this paper the problem is considerably simplified. When the edge energy, ***ϵ***, needed to form a monomolecular layer is large, the free energy gained by packing the loops in a flat surface is appreciable. Hence embryos or nuclei with different step heights **l**_1_ **l**_2_, … **l***_v_*, are energetically improbable compared to nuclei whose step elements have the same step height. Then in this case it is clearly justified to treat nuclei which may be characterized by a single step height. But it is only when ***ϵ*** is a large quantity that the distribution of step heights in primary critical nuclei need to be considered, for it is only then that the step height of the crystal will be determined by the step height of the critical primary nucleus, so that the distribution in step heights of the primary nuclei control the distribution in step heights of the crystals. If ***ϵ*** is a smaller quantity the distribution of the step heights of the crystals will be independent of that of the primary nuclei, and only the total nucleation rate is of interest. The total nucleation rate is given by eq (A-15) in any case.

### 5.2. Equilibrium Rate of Formation of Mono-molecular Nuclei on a Crystal Face

We wish to calculate the equilibrium nucleation rate of monomolecular nuclei on a crystal face. This problem is similar to the one treated in the previous appendix, except that the free energy surface is different. In particular, the activated nucleus is reached in a single elementary process from the supercooled liquid. It will be assumed that the nucleus can contain ***v*** step elements where ***v*** can possess the values

0,1,2,… (B-1)

The free energy required to form a nucleus of ***v*** step elements is

$$\begin{array}{ll} \Delta \phi_{0}^{\prime }=0, & v=0\\ \Delta \phi_{v}^{\prime }=\Delta {\phi^{\prime }}^{‡}-(v-1)E, & v=1,2,\ldots \end{array}$$ (B-2)

Then the activated state is at ***v***=1, where $\Delta \phi_{1}^{\prime }=\Delta {\phi^{\prime }}^{‡}$. The same method of derivation will be used as in appendix 5.1. Then the rate at which nuclei with ***v*** step elements become nuclei with (***v***+1) step elements is

$$\eta_{v}\left(\frac{kT}{h}\right)e^{-\Delta F*/kT}\;\exp\;\left[-\frac{\left(\Delta \phi_{v+1}^{\prime }-\Delta \phi_{v}^{\prime }\right)}{2kT}\right],$$ (B-3)

whereas the rate of the backward reaction is

$$\eta_{v+1}\left(\frac{kT}{h}\right)e^{-\Delta F*/kT}\;\exp\;\left[+\frac{\left(\Delta \phi_{v+1}^{\prime }-\Delta \phi_{v}^{\prime }\right)}{2kT}\right].$$ (B-4)

Here *η_v_* is the number of nuclei per unit volume with ***v*** step elements. The net rate of nucleation per unit volume, **r**, is

$$r=\left(\frac{kT}{h}\right)e^{-\Delta F*/kT}\left\{\eta_{v}\;\exp\left[\frac{-(\Delta \phi_{v+1}^{\prime }-\Delta \phi_{v}^{\prime })}{2kT}\right]-\eta_{v+1}\;\exp\left[\frac{\left(\Delta \phi_{v+1}^{\prime }-\Delta \phi_{v}^{\prime }\right)}{2kT}\right]\right\}.$$ (B-5)

If the rate is an equilibrium rate, **r** does not depend on ***v.*** Combining (B-2) with (B-5)

$$\begin{array}{l} r=\frac{kT}{h}e^{-\Delta F*/kT}\left\{\eta_{v}e^{E/2kT}-\eta_{v+1}e^{-E/2kT}\right\}\\ v=1,2,\ldots \end{array}$$ (B-6)

and

$$r=\left(\frac{kT}{h}\right)e^{-\Delta F*/kT}\{\eta_{0}e^{-\Delta {\phi^{\prime }}^{‡}/2kT}-\eta_{1}e^{\Delta {\phi^{\prime }}^{‡}/2kT}\}.$$ (B-7)

From eq (B-6)

$$\eta_{v}=\eta_{1}e^{+vE/kT}-r\left(\frac{h}{kT}\right)e^{\Delta F*/kT}\left\{\frac{\left[e^{vE/kT}-1\right]}{2\;\sinh\;E/2kT}\right\}.$$ (B-8)

Since we are concerned with the equilibrium rate, *η_v_* must be bounded as ***v***→∞. In order that this is satisfied

$$r=2\eta_{1}\left(\frac{kT}{h}\right)e^{-\Delta F*/kT}\sinh\;\left(\frac{E}{2kT}\right).$$ (B-9)

Combining eq (B-7) and (B-9) we may solve for **r:**

$$\begin{array}{l} r=\eta_{0}\left(\frac{kT}{h}\right)e^{-\Delta F*/kT}\;e^{-\Delta {\phi^{\prime }}^{‡}/kT}\\ \;\left\{\frac{2\sinh\;(E/2kT)}{1+2e^{-\Delta {\phi^{\prime }}^{‡}/2kT}\;\sinh\;(E/2kT)\;}\right\} \end{array}$$ (B-10)

This then is the desired equilibrium rate of formation of these nuclei.

Three points in this derivation are worthy of comment. First the derivation in this appendix differs from that in appendix 5.1 in that the activated state is attained by a single elementary process. Then the expression obtained in eq (B-10) must be applied with due caution since more detailed knowledge of the nucleation process is assumed than in the previous appendix. The second point is that by solving for *η_v_* from eqs (B-8), (B-9), and (B-10) it is found that

$$\eta_{v}=\frac{\eta_{0}e-\Delta {\phi^{\prime }}^{‡}/kT}{1+2\exp\{-\Delta {\phi^{\prime }}^{‡}/2kT\}\;\sinh\;(E/2kT)}.$$ (B-11)

Thus *η_v_* is independent of ***v***, and does not vanish as ***v*** becomes infinitely large. This raises the question of whether a very large time is required before the equilibrium rate is achieved so that the transient rate cannot be neglected. This question can be answered by a solution of the time dependent problem. It can be shown for the case treated here that the equilibrium rate is closely approached after the number of nuclei with a few step elements have nearly reached the value found in eq (B-11). Thus the equilibrium rate derived in eq (B-10) is applicable to the case at hand. Thirdly in both appendixes 5.1. and 5.2. it has been assumed tacitly that nuclei can be initiated at only one point in the polymer chain. If this assumption is taken into account in an appropriate manner, a numerical factor will be introduced into the nucleation rates obtained. This factor will almost certainly be less than 10^4^.

Equation (B-10) is first used in section 3.2. to describe the formation of a primary crystallite through monomolecular accretion from a critical-sized loop-type nucleus. It is later used to describe the growth of a crystallite in section 3.3., i.e., $\Delta {\phi^{\prime }}^{‡}\to \Delta \phi_{g}^{‡}$. Since in sections 3.2. and 3.3. the quantity **r***d***l** is taken as the rate of formation of nuclei with step heights between **l** and **l**+*d***l**, the quantity *η*_0_ will contain a normalization constant *C*, which arises because the rates of more than one competing process are considered.

## References

[1] Keller A, O’Connor A (1958). Discussions. Faraday Soc.

[2] Keller A (1957). Phil Mag.

[3] Till PH (1957). J Polymer Sci.

[4] Fischer EW (1957). Z Naturforsch.

[5] Storks KH (1938). J Am Chem Soc.

[6] Hoffman JD, Weeks JJ, Murphey WM (1959). J Research NBS.

[7] Mandelkern L (1956). Chem Rev.

[8] Turnbull D, Fisher JC (1949). J Chem Phys.

[9] Mandelkern L, Quinn FA, Flory PJ (1954). J Appl Phys.

[10] Hoffman JD (1958). J Chem Phys.

[b11-jresv64an1p73_a1b] 11J. I. Lauritzen, Jr. (to be publis hed).

[12] Bunn CW (1939). Trans Faraday Soc.

[13] Mandelkern L (1955). J Appl Phys.

[14] Quinn FA, Mandelkern L (1958). J Am Chem Soc.

[15] Flory PJ (1958). Principles of polymer chemistry.

[16] Thomas DG, Staveley LAK (1952). J Chem Soc.

[17] Mandelkern L, Quinn FA

[18] Mandelkern L (1959). SPE Journal.

[19] Price FP J Polymer Sci.

